# Association of the inflammation-related proteome with dementia development at older age: results from a large, prospective, population-based cohort study

**DOI:** 10.1186/s13195-022-01063-y

**Published:** 2022-09-09

**Authors:** Kira Trares, Megha Bhardwaj, Laura Perna, Hannah Stocker, Agnese Petrera, Stefanie M. Hauck, Konrad Beyreuther, Hermann Brenner, Ben Schöttker

**Affiliations:** 1 Network Aging Research, Heidelberg University, Bergheimer Straße 20, 69115 Heidelberg, Germany; 2 Division of Clinical Epidemiology and Aging Research, German Cancer Research Center, Im Neuenheimer Feld 581, 69120 Heidelberg, Germany; 3 Medical Faculty, Heidelberg University, Im Neuenheimer Feld 672, 69120 Heidelberg, Germany; 4 Department of Translational Research in Psychiatry, Max Planck Institute of Psychiatry, Kraepelinstraße 2-10, 80804 Munich, Germany; 5 Division of Mental Health of Older Adults, Department of Psychiatry and Psychotherapy, University Hospital, LMU Munich, 80336 Munich, Germany; 6 Research Unit Protein Science and Metabolomics and Proteomics Core Facility, Helmholtz Zentrum Munich - German Research Center for Environmental Health, Heidemannstraße 1, 80939 Munich, Germany

**Keywords:** Inflammation, Biomarker, Cohort study, Dementia, Alzheimer’s disease, Vascular dementia

## Abstract

**Background:**

Chronic inflammation is a central feature of several forms of dementia. However, few details on the associations of blood-based inflammation-related proteins with dementia incidence have been explored yet.

**Methods:**

The Olink Target 96 Inflammation panel was measured in baseline serum samples (collected 07/2000–06/2002) of 1782 older adults from a German, population-based cohort study in a case-cohort design. Logistic regression models were used to assess the associations of biomarkers with all-cause dementia, Alzheimer’s disease, and vascular dementia incidence.

**Results:**

During 17 years of follow-up, 504 participants were diagnosed with dementia, including 163 Alzheimer’s disease and 195 vascular dementia cases. After correction for multiple testing, 58 out of 72 tested (80.6%) biomarkers were statistically significantly associated with all-cause dementia, 22 with Alzheimer’s disease, and 33 with vascular dementia incidence. We identified four biomarker clusters, among which the strongest representatives, CX3CL1, EN-RAGE, LAP TGF-beta-1, and VEGF-A, were significantly associated with dementia endpoints independently from other inflammation-related proteins. CX3CL1 (odds ratio [95% confidence interval] per 1 standard deviation increase: 1.41 [1.24–1.60]) and EN-RAGE (1.41 [1.25–1.60]) were associated with all-cause dementia incidence, EN-RAGE (1.51 [1.25–1.83]) and LAP TGF-beta-1 (1.46 [1.21–1.76]) with Alzheimer’s disease incidence, and VEGF-A (1.43 [1.20–1.70]) with vascular dementia incidence. All named associations were stronger among *APOE* ε4-negative subjects.

**Conclusion:**

With this large, population-based cohort study, we show for the first time that the majority of inflammation-related proteins measured in blood samples are associated with total dementia incidence. Future studies should concentrate not only on single biomarkers but also on the complex relationships in biomarker clusters.

**Supplementary Information:**

The online version contains supplementary material available at 10.1186/s13195-022-01063-y.

## Introduction

Dementia is a major challenge for global public health and social care systems. As the number of dementia cases increases with rising life expectancy, it has been estimated that almost 75 million people worldwide will live with dementia in 2030 [1]. Therefore, research on how to prevent or delay the onset of dementia is one of the major challenges globally [2].

Inflammation likely plays a key role in the development and progression of dementia [3]. In the brain, inflammatory processes are a defence mechanism against infection, toxins, or injury. Persistent systemic inflammation, occurring outside the central nervous system (CNS), might disrupt the equilibrium of pro- and anti-inflammatory signalling. Released products can then cross the blood-brain barrier and lead to neuroinflammation. In neuroinflammation, microglia and astrocytes are activated and release various pro-inflammatory products causing chronic inflammation and neurodegeneration [4, 5]. Several studies have found increased levels of pro-inflammatory cytokines and proteins like interleukin-6 (IL-6), interleukin-1β (IL-1β), C-reactive protein (CRP), or α1-antichymotrypsin (α1-AT) to be associated with the onset of all-cause dementia [6, 7]. Further studies also revealed that CRP, IL-1ß, IL-2, IL-4, IL-6, IL-8, IL-10, IL-12, IL-18, monocyte protein-1 (MCP-1), MCP-3, interferon-γ-inducible protein 10 (IP-10), and tumour necrosis factor α (TNF-α) are associated with the incidence of Alzheimer’s disease, the most common form of dementia, accounting for 60–80% of all cases [8, 9]. However, longitudinal studies on the association between biomarkers of the inflammation-related proteome and dementia are scarce [10].

As biomarkers can be measured at an early stage of dementia, they have the potential to be used for an early diagnosis [11, 12]. Furthermore, identified new biomarkers could deepen our understanding of the pathogenetic processes leading to dementia and might represent novel drug targets [13, 14]. The current challenge of biomarker research in dementia in general and distinct forms is to find reliable diagnostic and predictive biomarkers easily accessible in fluids like blood [15, 16].

This study aims for the first time to identify blood-based biomarkers from a set of 92 inflammatory biomarkers as risk factors for all-cause dementia, Alzheimer’s disease, or vascular dementia incidence in a large, prospective cohort study with a 17-year follow-up.

## Methods

### Study population

We conducted a case-cohort study based on the ESTHER study (Epidemiologische Studie zu Chancen der Verhütung, Früherkennung und optimierten Therapie chronischer Erkrankungen in der älteren Bevölkerung [German]). In this prospective cohort study implemented in Saarland, Germany, 9940 women and men aged 50 to 75 years at baseline were recruited during a general health checkup by their general practitioners between 2000 and 2002. Besides an age of 50–75 years, the inclusion criteria for the ESTHER study were physical and mental ability to participate in the study as well as knowledge of the German language. Participants were followed up concerning the incidence of major diseases and mortality 2, 5, 8, 11, 14, and 17 years after baseline. For details, see Löw et al. [17]. The sociodemographic baseline characteristics and common prevalent chronic diseases were similarly distributed in the respective age categories as in the German National Health Survey, a representative sample of the German population [17].

### Dementia ascertainment

Information about a dementia diagnosis was collected during the 14- and 17-year follow-ups of the ESTHER study. The median follow-up time was 16.3 years (interquartile range: 13.5–17.0 years), and the maximum was 19.4 years due to the 2-year period of baseline recruitment. In brief, the collection of dementia diagnoses included sending standardized questionnaires to the study participants’ general practitioners (GPs). Participants who had dropped out during previous follow-ups due to ill health or had died were included in the dementia ascertainment through the GPs as well. If the GPs were aware of a dementia diagnosis for their patients, they were asked to provide a date of diagnosis and all available medical records documenting a dementia diagnosis. The latter included records from neurologists, psychiatrists, memory clinics, or other specialized providers. If the GP provided a mixed dementia diagnosis, available medical records were screened for an underlying Alzheimer’s disease or vascular dementia background and considered as Alzheimer’s disease, vascular dementia, or both. The Alzheimer’s disease diagnosis guideline used in Germany during the follow-up period of the ESTHER study was the one of the National Institute on Aging and the Alzheimer’s Association [18].

### Biomarker measurements

Inflammation-related, blood-based proteins were measured from serum samples collected during the health checkup at baseline (2000–2002). Blood samples were sent to the study centre and stored at −80°C until biomarker measurements took place in three waves in March 2018, December 2018, and September 2020 (referred to as time points t1, t2, and t3 in the following). At the time of the measurements, 10–25 μl of serum was extracted from different aliquots that had been thawed twice and sent with dry ice to the laboratories, which analysed the samples with the Olink Target 96 Inflammation panel, Olink Proteomics, Uppsala, Sweden. At t1 and t2, samples were analysed in the laboratory of Olink Proteomics, Uppsala Science Park, SE-75183 Uppsala, Sweden. At t3, the measurements were performed in the Research Unit Protein Science, German Research Center for Environmental Health, Helmholtz Center Munich, Heidemannstraße 1, 80939 München, Germany.

The Olink panels are based on a proximity extension assay technology (PEA) [19, 20]. Details on the reliability and stability of the technology are described elsewhere [21]. In brief, oligonucleotide-labelled antibody probe pairs are allowed to bind to their respective target proteins in the samples. Only if two antibodies are in close proximity, a polymerase chain reaction (PCR) reporter sequence is formed by DNA polymerization. This sequence is detected and quantified using high-throughput real-time quantitative PCR (qPCR) (Fluidigm® Biomark^TM^ HD system). The Olink Target 96 Inflammation panel allows the measurement of 92 biomarkers per sample. A list of all biomarkers of this panel is displayed in Supplemental Table 1.

At t1, t2, and t3, 22, 15, and 5 plates were used, respectively. To avoid batch effects, cases and controls were randomly distributed across plates and adjusted according to included interpolate controls. The average intra-assay coefficient of variance among all 92 measured biomarkers was 7%, 4%, and 3% at t1, t2, and t3, respectively. The average inter-assay coefficient of variance was 12%, 10%, and 10% at t1, t2, and t3, respectively. Furthermore, the quality of each serum sample was assessed by Olink technology [22]. All samples were measured successfully, and the number of quality control warnings was below 4% in all three timepoints. Of the 1435 randomly selected controls and 393 incident dementia cases, 46 serum samples of participants were excluded due to a quality control warning by Olink.

Protein levels are reported as Normalized Protein eXpression (NPX) values, a relative quantification unit logarithmically related to protein concentration. The number of samples with values below the lower limit of detection (LOD) varied strongly by biomarker and is shown in Supplemental Table 1. In total, 20 biomarkers with > 25% of the values below LOD were excluded from all analyses (grey-shaded biomarkers in Supplemental Table 1). Thereby, 72 out of the 92 biomarkers were considered evaluable markers. Biomarker values below the LOD were replaced by LOD/√2. The normalization of raw data was conducted with the R (R Core Team, 2020, version 3.6.3) package “OlinkAnalyze”, developed and maintained by the Olink Proteomics Data Science Team [23]. To normalize data from three different measurement points (t1, t2, and t3), reference sample normalization was used based on 17 and 16 bridging samples between each two measurement points (t1–t3 and t2–t3, pair-wise bridging). For details of the procedure, we refer to the white paper of OLINK Proteomics [22].

### Covariate assessment

Data on sex, age, education, body mass index (BMI), physical activity, and life-time history of depression were collected during baseline assessment through a standardized self-administered questionnaire. The history of coronary heart disease (CHD) and diabetes mellitus were obtained from physician diagnoses. Furthermore, anti-diabetic drugs reported by the GP were used to complement diabetes mellitus diagnoses. Participants were considered to have cardiovascular disease (CVD) based upon CHD diagnoses from GPs or self-reported history of myocardial infarction, stroke, pulmonary embolism, or revascularization of coronary arteries. TaqMan single-nucleotide polymorphism (SNP) genotyping assays were used to determine apolipoprotein E (*APOE*) genotypes. More precisely, genotypes were analysed in an endpoint allelic discrimination using a PRISM 7000 Sequence detection system (Applied Biosystems) [24].

### Inclusion and exclusion criteria

**Fig. 1:**
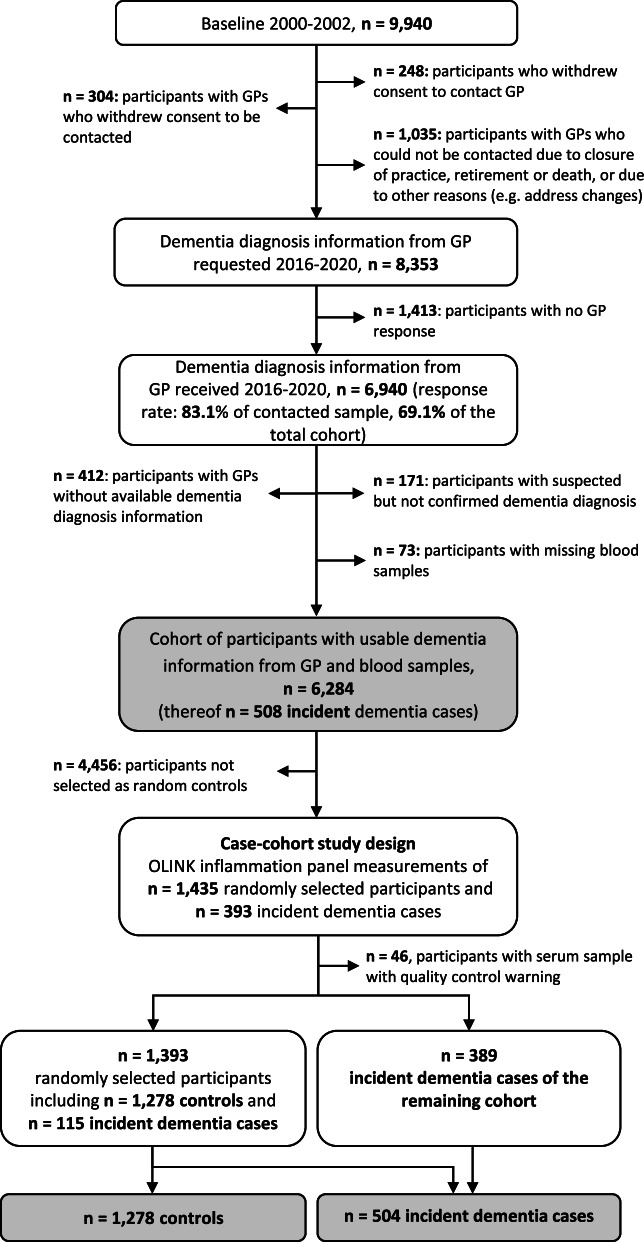
Flowchart of dementia ascertainment during the 14- and 17-year follow-up of the ESTHER study and selection of the study population for this research project. Abbreviations: GP, general practitioner

The selection of study participants from the ESTHER cohort for this case-cohort analysis is shown in Fig. 1. ESTHER participants were eligible for selection as cases or random controls. Participants were excluded if they withdrew consent to contact the GP (*n*=248), if the GP withdrew consent to be contacted (*n*=304), or if the GP could not be reached anymore (*n*=1035). Thereupon, dementia diagnosis information was requested for 8353 participants and received for 6940 participants (response rate: 83.1%). Moreover, participants were excluded if dementia incidence status could not be ascertained by GP questionnaires (either diagnosis was not available (*n*=412) or not confirmed (*n*=171)) or if blood samples were not available (*n*=73). Thus, information from 6284 participants was available for analyses. Except for their age, participants with available dementia information and blood samples and those who could not be used for analyses had comparable baseline characteristics (Supplemental Table 2). Fewer included than excluded participants were in the oldest age group of 70 to 75 years (13.8% compared to 19.0%) reflecting the challenges to obtain dementia information about deceased study participants from their GPs.

Olink inflammation panel measurements were performed in a case-cohort design among 1435 randomly selected study participants and all incident dementia cases of the rest of the cohort (*n*=393). To check if the random selection was successful, we compared the baseline characteristics of selected and non-selected controls and observed no substantial differences between the groups (Supplemental Table 3). After excluding participants with quality control warning, 389 incident dementia cases and 1393 randomly selected participants were available. As the randomly selected participants included 115 incident dementia cases, the study population comprised 504 participants with incident dementia and 1278 randomly selected controls.

### Statistical analyses

First, to describe factors associated with dementia risk, categorized baseline characteristics of all-cause dementia cases and controls were compared using the *χ*^2^-test. Second, odds ratios (ORs) for all-cause dementia were estimated with a multivariate logistic regression model, including all baseline characteristics shown in Table 1.

In a univariate, descriptive analysis, the median and interquartile range (IQR) of all inflammation-related protein levels of all-cause dementia, Alzheimer’s disease, and vascular dementia cases were separately compared with those of controls, using the Wilcoxon rank sum test. Additionally, in a multivariate approach, the ORs per one standard deviation (SD) increase of each inflammation-related protein were assessed separately with each outcome (all-cause dementia, Alzheimer’s disease, and vascular dementia incidence) in logistic regression models adjusted for potential confounders. In models for Alzheimer’s disease incidence, study participants with other (e.g. vascular dementia) or unknown dementia forms were excluded. The same was applied for the outcome vascular dementia incidence by excluding Alzheimer’s disease and other non-vascular dementia cases. The models were adjusted for age, sex, education, physical activity, BMI, CVD, diabetes, depression, and *APOE* genotype. All variables were used as categorical variables, as described in Table 1, except age, which was modelled continuously. The covariates were selected because they were statistically significantly associated with all-cause dementia, Alzheimer’s disease, or vascular dementia in a previous analysis with the ESTHER study participants [25]. Statistical test results were corrected for multiple testing by the Benjamini and Hochberg method for all tests carried out for one outcome [26]. A false discovery rate (FDR) < 0.05 was applied as the threshold for statistical significance. In a sensitivity analysis, the multivariate logistic regression model was repeated using weighted Cox regression, according to Barlow et al. [27].

We further aimed to identify those inflammation-related proteins whose association with a dementia outcome was independent of other inflammatory biomarkers. Therefore, all biomarkers, which were significantly associated with a dementia endpoint after FDR correction, were tested for the independence of the association by forward elimination. Only biomarkers having the strongest, independent, positive association with the outcome entered the regression model with the threshold for statistical significance of *p*<0.05 in the following logistic regression analysis. Moreover, biomarker clusters were built and named based on the identified independent biomarkers. All other biomarkers of the Olink inflammation panel, which were highly correlated (Spearman’s correlation coefficient *r* > 0.5) [28] with an independent biomarker, were put in its cluster. One biomarker might be in more than one cluster. We favoured this statistical approach over a principal component analysis because it has a higher transparency and is easier to reproduce by others, its results are easier to interpret, and the associations of the biomarkers with the dementia outcomes are being acknowledged in the decision about the number of clusters.

The associations of the independent biomarkers with dementia endpoints were further analysed in subgroup analyses based on age, sex, obesity, diabetes, history of CVD, and *APOE* ε4 polymorphism. These factors were selected a priori because they are important dementia risk factors and determinants of inflammation [29]. Apart from this, interaction terms were tested. In addition, the dose-response relationships between the independent biomarkers and dementia endpoints were assessed with restricted cubic spline curves [30].

Several sensitivity analyses were performed. To check for potential reverse causality, the associations between the independent biomarkers and dementia endpoints were analysed stratified by time of diagnosis (in the first 10 years of follow-up vs later years). Competing risk of death was examined by excluding subjects without dementia diagnosis who died before their 80th birthday, the average life expectancy of the cohort’s population. Fractional polynomials with first-order terms were used to determine each biomarker’s best fitting function with the outcomes [31]. The linear function was the best fitting one for almost all biomarkers (67 of 72). Since the low number of biomarkers with deviations in the best fitting function from linearity (6.9%) could have resulted from multiple testing, all were modelled linearly. In a sensitivity analysis, the multivariate logistic regression analysis was repeated with the five non-linear biomarkers modelled with their best fitting function. Finally, to examine the impact of persons with a potential acute infection on the overall results, subjects with C-reactive protein (CRP) levels > 20 mg/L were excluded.

To our knowledge, missing values of covariates were missing at random. The highest proportion of missing values was found for *APOE* polymorphism (7.5%). Thus, multiple imputation was used to impute missing values. Variables shown in Table 1 were used for the imputation model. Twenty data sets were imputed with the Markov Chain Monte Carlo (MCMC) method separately by sex with the SAS procedure PROC MI. All analyses were performed based on those 20 datasets with the SAS procedure PROC MIANALYZE.

Statistical tests were two-sided using an alpha level of 0.05. All statistical analyses were conducted with the Statistical Analysis System (SAS, version 9.4, Cary, North Carolina, USA).

## Results

Table 1 shows the baseline characteristics of 504 cases with incident dementia from any cause and 1278 controls. The *χ*^2^ test revealed significant differences between cases and controls in terms of age, physical activity, CVD, diabetes, life-time history of depression, and *APOE* genotype. In the multivariate logistic regression analysis, including all baseline characteristics shown in Table 1, CVD and diabetes lost statistical significance, but a trend towards an increased all-cause dementia risk could still be seen in the OR point estimates. Male sex became statistically significantly associated with all-cause dementia incidence while age, physical activity (inversely), depression with current pharmacotherapy, and the APOE genotypes ɛ2/ɛ4, ɛ3/ɛ4, and ɛ4/ɛ4 remained significantly associated with all-cause dementia incidence. The APOE genotype ɛ2/ɛ3 as well as a life-time history of depression without current pharmacotherapy were not associated with all-cause dementia. The APOE genotype ɛ2/ɛ2, school education ≥ 10 years and a BMI ≥ 25 kg/m^2^ suggested inverse associations with dementia but were not statistically significant.

**Table 1:** Baseline characteristics of included study participants (*n*=1782) and their association with all-cause dementia

Baseline characteristics	***n*** (%)	All-cause dementia cases (***n***=504)	Controls (***n***=1278)	***χ***^**2**^ test ***p***-value	Multivariate odds ratio (95% CI)^c^
**Age (years)**				**< 0.0001**	
50–64	956 (53.65)	154 (30.56)	802 (62.75)		1.00 Ref.
65–69	458 (25.70)	157 (31.15)	301 (23.55)		**2.57 (1.96**–**3.37)**
70–75	368 (20.65)	193 (38.29)	175 (13.69)		**5.37 (4.03**–**7.15)**
**Sex**				0.2486	
Female	965 (54.15)	262 (51.98)	703 (55.01)		1.00 Ref.
Male	817 (45.85)	242 (48.02)	575 (44.99)		**1.28 (1.01**–**1.63)**
**Education (years)**				0.0868	
≤ 9	1344 (77.42)	391 (80.79)	953 (76.12)		1.00 Ref.
10–11	216 (12.44)	48 (9.92)	168 (13.42)		0.84 (0.58–1.23)
≥ 12	176 (10.14)	45 (9.30)	131 (10.46)		0.93 (0.62–1.38)
**Physical activity**^a^				**< 0.0001**	
Inactive	383 (21.54)	150 (29.82)	233 (18.27)		1.00 Ref.
Low	814 (45.78)	220 (43.74)	594 (46.59)		**0.65 (0.49**–**0.86)**
Medium or high	581 (32.68)	133 (26.44)	448 (35.14)		**0.60 (0.44**–**0.83)**
**BMI (kg/m**^**2**^**)**				0.5708	
< 25	478 (26.91)	144 (28.63)	334 (26.24)		1.00 Ref.
25–<30	832 (46.85)	228 (45.33)	604 (47.45)		0.85 (0.65–1.12)
≥30	466 (26.24)	131 (26.04)	335 (26.32)		0.85 (0.62–1.17)
**CVD**^b^				**< 0.0001**	
No	1373 (77.05)	350 (69.44)	1023 (80.05)		1.00 Ref.
Yes	409 (22.95)	154 (30.56)	255 (19.95)		1.20 (0.92–1.56)
**Diabetes**				**0.0001**	
No	1469 (83.61)	386 (78.14)	1083 (85.75)		1.00 Ref.
Yes	288 (16.39)	108 (21.86)	180 (14.25)		1.29 (0.96–1.74)
**Life-time history of depression**				**0.0225**	
No	1527 (85.69)	427 (84.72)	1100 (86.07)		1.00 Ref.
Yes, without current pharmacotherapy	184 (10.33)	47 (9.33)	137 (10.72)		1.01 (0.69–1.49)
Yes, with current pharmacotherapy	71 (3.98)	30 (5.95)	41 (3.21)		**2.26 (1.33**–**3.85)**
**APOE genotypes**^d^				**< 0.0001**	
ɛ2/ɛ2	18 (1.09)	1 (0.22)	17 (1.42)		0.25 (0.04–1.47)
ɛ2/ɛ3	238 (14.43)	57 (12.58)	181 (15.13)		1.06 (0.75–1.52)
ɛ2/ɛ4	55 (3.34)	22 (4.86)	33 (2.76)		**2.77 (1.52**–**5.06)**
ɛ3/ɛ3	929 (56.34)	218 (48.12)	711 (59.45)		1.00 Ref.
ɛ3/ɛ4	379 (22.98)	135 (29.80)	244 (20.40)		**1.79 (1.35**–**2.37)**
ɛ4/ɛ4	30 (1.82)	20 (4.42)	10 (0.84)		**7.15 (3.18**–**16.08)**

Numbers printed in bold are statistically significant (*p* < 0.05)

*Abbreviations*: *CI*, confidence interval; *BMI*, body mass index; *CVD*, cardiovascular disease; *APOE*, apolipoprotein E

^a^“Inactive” was defined by < 1 h of vigorous or < 1 h light physical activity per week. “Medium or high” was defined by ≥ 2 h of vigorous and ≥ 2 h of light physical activity/week. All other amounts of physical activity were grouped into the category “Low”

^b^CVD was defined as coronary artery disease or a self-reported history of myocardial infarction, stroke, pulmonary embolism, or revascularization of coronary arteries

^c^Results of multivariate logistic regression model for all-cause dementia including all variables shown in this table (imputed dataset)

^d^APOE genotypes could not be ascertained for 7.5% of the participants due to problems with DNA extraction in the process of analyses

Among the included 504 all-cause dementia cases, 163 and 195 participants developed Alzheimer’s disease and vascular dementia, respectively. The medians of all inflammation-related protein levels of all-cause dementia, Alzheimer’s disease, and vascular dementia cases were separately compared with those of controls (Supplemental Tables 4-6). In this univariate analysis, *n* = 60, *n* = 51, and *n* = 52 biomarker levels of the Olink inflammation panel were significantly increased in all-cause dementia, Alzheimer’s disease, and vascular dementia cases, respectively (FDR < 0.05).

Tables 2, 3, and 4 show the multivariate logistic regression model results for those 58, 22, and 33 biomarkers, significantly associated with all-cause dementia, Alzheimer’s disease, and vascular dementia incidence, respectively, after FDR correction. Supplemental Tables 7–9 show the non-significant ones. The associations’ strengths were comparable and ranged for the various biomarker-outcome associations from OR point estimates of 1.12 to 1.51 per 1 SD increase.

**Table 2:** Associations of significantly associated Olink Biomarker levels with all-cause dementia incidence. For associations of not significantly associated biomarkers, see Supplemental Table 6

Olink Biomarker	Value of 1 SD	All-cause dementia (***n***=504 cases)		
		OR (95% CI) per 1 SD^*^	***p***-value per 1 SD	FDR corrected ***p***-value^†^
ADA	0.635	1.17 (1.05-1.32)	0.0055	0.0098
AXIN1	1.140	1.14 (1.02-1.28)	0.0257	0.0352
CASP-8	1.364	1.15 (1.02-1.29)	0.0194	0.0279
CCL3	1.508	1.14 (1.02-1.27)	0.0259	0.0352
CCL4	1.099	1.19 (1.06-1.33)	0.0032	0.0066
CCL11	0.697	1.29 (1.14-1.46)	0.0001	0.0003
CCL19	1.199	1.17 (1.05-1.32)	0.0063	0.0108
CCL20	1.540	1.18 (1.05-1.32)	0.0041	0.0082
CCL23	0.732	1.29 (1.14-1.46)	<0.0001	0.0003
CCL25	0.763	1.17 (1.03-1.32)	0.0122	0.0187
CCL28	0.548	1.27 (1.13-1.43)	<0.0001	0.0003
CD5	0.523	1.26 (1.12-1.42)	0.0002	0.0007
CD6	0.757	1.19 (1.05-1.34)	0.0048	0.0093
CD40	0.734	1.20 (1.07-1.36)	0.0022	0.0050
CD244	0.587	1.38 (1.22-1.57)	<0.0001	0.0003
CDCP1	0.894	1.20 (1.06-1.36)	0.0030	0.0064
CSF-1	0.425	1.24 (1.09-1.41)	0.0013	0.0032
CST5	0.698	1.17 (1.04-1.32)	0.0112	0.0179
**CX3CL1**	**0.669**	**1.41 (1.24-1.60)**	**<0.0001**	**0.0003**
CXCL1	0.901	1.17 (1.05-1.32)	0.0065	0.0109
CXCL5	0.957	1.33 (1.17-1.51)	<0.0001	0.0003
CXCL6	0.848	1.28 (1.14-1.44)	0.0001	0.0003
CXCL9	0.953	1.19 (1.05-1.34)	0.0052	0.0096
CXCL10	0.953	1.16 (1.03-1.30)	0.0138	0.0207
CXCL11	1.051	1.18 (1.05-1.33)	0.0051	0.0096
DNER	0.488	1.36 (1.20-1.55)	<0.0001	0.0003
**EN-RAGE**	**1.307**	**1.41 (1.25-1.60)**	**<0.0001**	**0.0003**
FGF-19	1.089	1.21 (1.08-1.35)	0.0013	0.0032
Flt3L	0.629	1.21 (1.07-1.36)	0.0018	0.0042
GDNF	0.506	1.16 (1.03-1.30)	0.0149	0.0219
HGF	0.719	1.34 (1.18-1.52)	<0.0001	0.0003
IL-7	0.798	1.14 (1.02-1.28)	0.0246	0.0347
IL-10	0.863	1.20 (1.07-1.35)	0.0023	0.0050
IL-18	0.763	1.33 (1.17-1.50)	<0.0001	0.0003
IL-10RA	0.788	1.12 (1.01-1.25)	0.0362	0.0461
IL-10RB	0.533	1.29 (1.14-1.46)	0.0001	0.0003
IL-15RA	0.359	1.22 (1.09-1.38)	0.0009	0.0026
IL-18R1	0.602	1.27 (1.13-1.44)	0.0001	0.0003
LAP TGF-beta-1	0.574	1.37 (1.21-1.55)	<0.0001	0.0003
LIF-R	0.503	1.37 (1.21-1.56)	<0.0001	0.0003
MCP-2	0.769	1.18 (1.05-1.33)	0.0069	0.0113
MCP-4	0.927	1.23 (1.08-1.39)	0.0015	0.0036
MMP-10	0.761	1.22 (1.08-1.37)	0.0010	0.0028
NT-3	0.544	1.24 (1.10-1.39)	0.0003	0.0009
OPG	0.609	1.39 (1.22-1.58)	<0.0001	0.0003
PD-L1	0.600	1.30 (1.15-1.47)	<0.0001	0.0003
SCF	0.624	1.15 (1.01-1.29)	0.0281	0.0375
SIRT2	1.157	1.13 (1.01-1.27)	0.0371	0.0461
ST1A1	1.304	1.13 (1.01-1.27)	0.0365	0.0461
STAMBP	0.833	1.18 (1.05-1.32)	0.0056	0.0098
TGF-alpha	0.829	1.21 (1.08-1.37)	0.0011	0.0029
TNFRSF9	0.636	1.23 (1.09-1.39)	0.0006	0.0018
TNFSF14	1.064	1.16 (1.03-1.30)	0.0119	0.0186
TRAIL	0.513	1.31 (1.16-1.49)	<0.0001	0.0003
TRANCE	0.753	1.14 (1.01-1.28)	0.0344	0.0450
TWEAK	0.647	1.35 (1.19-1.53)	<0.0001	0.0003
VEGF-A	0.794	1.40 (1.24-1.59)	<0.0001	0.0003
uPA	0.604	1.36 (1.20-1.54)	<0.0001	0.0003

Abbreviations: *SD* standard deviation, *CI* confidence interval, *FDR* false discovery rate, For biomarker abbreviations, see Supplemental Table 1

All associations between Olink Biomarkers and the dementia outcome in this table are statistically significant after correction for multiple testing (FDR corrected p < 0.05). The lines for the most important biomarkers, CX3CL1 and EN-RAGE, which were also independently from other Olink biomarkers associated with the dementia outcome, are printed in bold. However, please note that this table shows their association with the outcome when they are put singularly in the multivariate logistic regression model

^*^ Multivariate logistic regression model adjusted for age (continuously), sex, education, physical activity, BMI (categorical), CVD, diabetes, depression, *APOE* genotype

^†^ *P*-values corrected for multiple testing by the Benjamini and Hochberg method

**Table 3:** Associations of significantly associated Olink Biomarker levels with Alzheimer’s disease incidence. For associations of not significantly associated biomarkers, see Supplemental Table 7

Olink Biomarker	Value of 1 SD	Alzheimer’s disease (***n***=163 cases)		
		OR (95% CI) per 1 SD^*^	***p***-value per 1 SD	FDR corrected ***p***-value^†^
CASP-8	1.364	1.31 (1.10-1.57)	0.0025	0.0156
CCL23	0.732	1.43 (1.17-1.75)	0.0004	0.0086
CCL28	0.548	1.36 (1.14-1.61)	0.0005	0.0086
CD6	0.757	1.30 (1.08-1.58)	0.0067	0.0254
CD244	0.587	1.39 (1.14-1.70)	0.0010	0.0120
CX3CL1	0.669	1.35 (1.10-1.65)	0.0034	0.0175
CXCL5	0.957	1.37 (1.12-1.68)	0.0023	0.0156
CXCL6	0.848	1.34 (1.11-1.62)	0.0026	0.0156
DNER	0.488	1.37 (1.12-1.68)	0.0025	0.0156
**EN-RAGE**	**1.307**	**1.51 (1.25-1.83)**	**<0.0001**	**0.0036**
HGF	0.719	1.36 (1.12-1.66)	0.0017	0.0153
IL-10RB	0.533	1.33 (1.08-1.63)	0.0066	0.0254
**LAP TGF-beta-1**	**0.574**	**1.46 (1.21-1.76)**	**0.0001**	**0.0036**
LIF-R	0.503	1.31 (1.08-1.60)	0.0062	0.0254
PD-L1	0.600	1.31 (1.09-1.57)	0.0034	0.0175
ST1A1	1.304	1.30 (1.08-1.57)	0.0062	0.0254
STAMBP	0.833	1.26 (1.06-1.51)	0.0108	0.0370
TGF-alpha	0.829	1.26 (1.05-1.52)	0.0140	0.0458
TRAIL	0.513	1.30 (1.06-1.59)	0.0104	0.0370
TWEAK	0.647	1.38 (1.13-1.69)	0.0016	0.0153
VEGF-A	0.794	1.32 (1.09-1.60)	0.0042	0.0202
uPA	0.604	1.40 (1.16-1.71)	0.0006	0.0086

Abbreviations: *SD* standard deviation, *CI* confidence interval, *FDR* false discovery rate; For biomarker abbreviations, see Supplemental Table 1

All associations between Olink Biomarkers and the dementia outcome in this table are statistically significant after correction for multiple testing (FDR corrected p < 0.05). The lines for the most important biomarkers, EN-RAGE and LAP TGF-beta-1, which were also independently from other Olink biomarkers associated with the dementia outcome, are printed in bold. However, please note that this table shows their association with the outcome when they are put singularly in the multivariate logistic regression model

^*^ Multivariate logistic regression model adjusted for age (continuously), sex, education, physical activity, BMI (categorical), CVD, diabetes, depression, *APOE* genotype

^†^ *P*-values corrected for multiple testing by the Benjamini and Hochberg method

**Table 4:** Associations of significantly associated Olink Biomarker levels with vascular dementia incidence. For associations of not significantly associated biomarkers, see Supplemental Table 8

Olink Biomarker	Value of 1 SD	Vascular dementia (***n***=195 cases)		
		OR (95% CI) per 1 SD^*^	***p***-value per 1 SD	FDR corrected ***p***-value^†^
CCL11	0.697	1.30 (1.09-1.56)	0.0042	0.0137
CCL23	0.732	1.24 (1.04-1.48)	0.0148	0.0347
CD5	0.523	1.32 (1.11-1.56)	0.0016	0.0091
CD244	0.587	1.39 (1.16-1.66)	0.0004	0.0072
CDCP1	0.894	1.25 (1.05-1.48)	0.0122	0.0313
CX3CL1	0.669	1.35 (1.13-1.61)	0.0011	0.0072
CXCL1	0.901	1.22 (1.04-1.43)	0.0151	0.0347
CXCL5	0.957	1.39 (1.16-1.67)	0.0004	0.0072
CXCL6	0.848	1.32 (1.11-1.57)	0.0018	0.0091
CXCL9	0.953	1.25 (1.06-1.47)	0.0089	0.0256
CXCL10	0.953	1.28 (1.09-1.50)	0.0029	0.0116
DNER	0.488	1.37 (1.13-1.65)	0.0010	0.0072
EN-RAGE	1.307	1.41 (1.18-1.68)	0.0001	0.0036
Flt3L	0.629	1.22 (1.03-1.45)	0.0226	0.0493
HGF	0.719	1.32 (1.11-1.58)	0.0019	0.0091
IL-7	0.798	1.24 (1.05-1.47)	0.0107	0.0296
IL-10	0.863	1.27 (1.09-1.48)	0.0017	0.0091
IL-18	0.763	1.36 (1.14-1.63)	0.0006	0.0072
IL-18R1	0.602	1.30 (1.09-1.55)	0.0034	0.0122
LAP TGF-beta-1	0.574	1.33 (1.12-1.57)	0.0011	0.0072
LIF-R	0.503	1.36 (1.14-1.63)	0.0007	0.0072
MCP-2	0.769	1.24 (1.04-1.47)	0.0154	0.0347
MCP-4	0.927	1.26 (1.05-1.51)	0.0114	0.0304
MMP-10	0.761	1.30 (1.10-1.54)	0.0022	0.0099
NT-3	0.544	1.26 (1.08-1.46)	0.0025	0.0106
OPG	0.609	1.38 (1.15-1.67)	0.0007	0.0072
PD-L1	0.600	1.26 (1.07-1.49)	0.0056	0.0175
TGF-alpha	0.829	1.23 (1.05-1.46)	0.0126	0.0313
TNFRSF9	0.636	1.26 (1.06-1.49)	0.0073	0.0219
TRAIL	0.513	1.32 (1.09-1.59)	0.0037	0.0127
TWEAK	0.647	1.36 (1.13-1.63)	0.0010	0.0072
**VEGF-A**	**0.794**	**1.43 (1.20-1.70)**	**0.0001**	**0.0036**
uPA	0.604	1.30 (1.09-1.55)	0.0034	0.0122

Abbreviations: *SD* standard deviation, *CI* confidence interval, *FDR* false discovery rate; For biomarker abbreviations, see Supplemental Table 1

All associations between Olink Biomarkers and the dementia outcome in this table are statistically significant after correction for multiple testing (FDR corrected p < 0.05). The line for the most important biomarker, VEGF-A, which is also independently from other Olink biomarkers associated with the dementia outcome, is printed in bold. However, please note that this table shows its association with the outcome when the biomarker is put singularly in the multivariate logistic regression model

^*^ Multivariate logistic regression model adjusted for age (continuously), sex, education, physical activity, BMI (categorical), CVD, diabetes, depression, *APOE* genotype

^†^ *P*-values corrected for multiple testing by the Benjamini and Hochberg method

In a sensitivity analysis, all biomarkers with strong associations (OR ≥ 1.30) were replicated with comparable strength of the associations with the dementia outcomes with weighted Cox regression (Supplemental Tables 10–12 for all-cause dementia, Alzheimer’s disease, and vascular dementia). However, for Alzheimer’s disease and vascular dementia, not all biomarkers, which were statistically significant in the logistic regression model, reached statistical significance in the weighted Cox regression model because the latter had lower statistical power. The differences between the results with weighted Cox regression and logistic regression were larger for biomarkers with weaker associations in the logistic regression (OR < 1.30) but with no clear direction (data not shown).

The forward selection revealed that only two (CX3CL1 and EN-RAGE), two (EN-RAGE and LAP TGF-beta-1), and one (VEGF-A) inflammation-related proteins were independently, positively associated with all-cause dementia, Alzheimer’s disease, and vascular dementia, respectively. The reason for the low number of independent inflammation biomarkers was mainly due to high inter-correlation. Overall, 18, 26, 16, and 28 biomarkers of the Olink inflammation panel had a Spearman’s *r* > 0.5 with CX3CL1, EN-RAGE, LAP TGF-beta 1, and VEGF-A, respectively (Supplemental Tables 13–17).

**Fig. 2:**
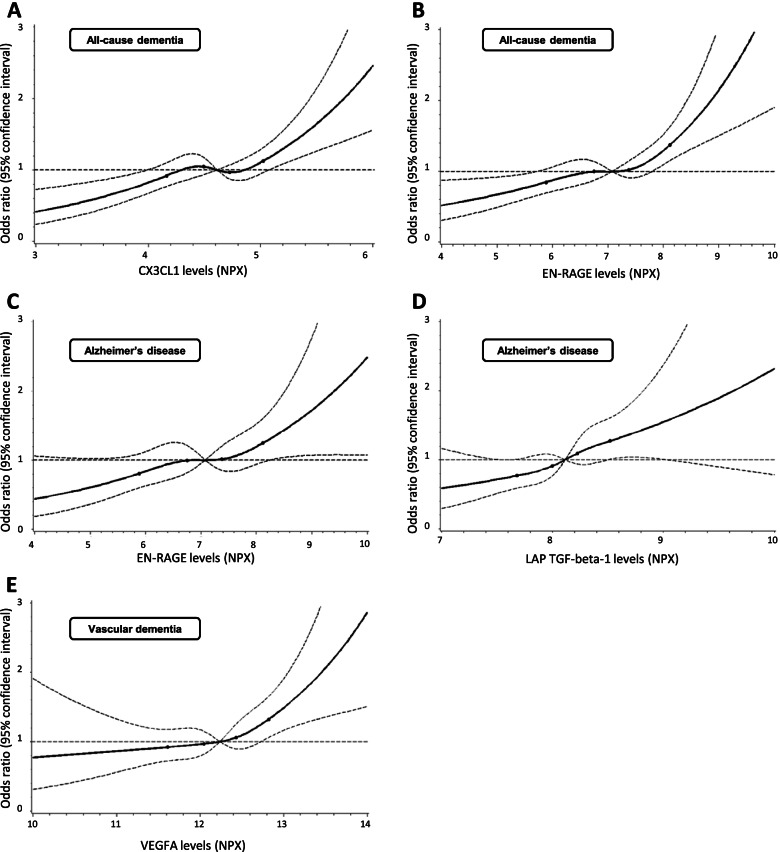
Association of all-cause dementia with **A** CX3CL1 and **B** EN-RAGE, Alzheimer’s disease with **C** EN-RAGE and **D** LAP TGF-beta-1, and vascular dementia with **E** VEGFA in a spline regression model adjusted for age (continuously), sex, education, physical activity, BMI (categorical), cardiovascular disease, diabetes, depression, and APOE genotype. Solid lines: estimation; dashed curved lines: 95% confidence interval limits; dashed horizontal line: reference line (hazard ratio = 1); dots: knots (20th, 40th, 60th, and 80th percentile). Abbreviations: NPX, Normalized Protein eXpression

When the two independent biomarkers for all-cause dementia were added simultaneously to the logistic regression models, the OR point estimates per 1 SD increase were attenuated but remained statistically significant (CX3CL1, OR [95% CI]: 1.29 [1.13–1.47], *p*=0.0002; EN-RAGE, OR [95% CI]: 1.31 [1.15–1.49], *p*<0.0001). This was also the case for the two independent biomarkers for Alzheimer’s disease (EN-RAGE, OR [95% CI]: 1.37 [1.10–1.68], *p*=0.0048; LAP TGF-beta-1, OR [95% CI]: 1.28 [1.04–1.58], *p*=0.0187). For vascular dementia, only one independent biomarker was included (VEGF-A, OR [95% CI]: 1.43 [1.20–1.70], *p*<0.0001). The dose-response curves of these five biomarker-dementia outcome associations are shown in Fig. 2. The risk of vascular dementia seems to start to increase only at higher VEGF-A levels (> 60th percentile). The other four biomarker-dementia associations show a more or less linear risk increase over the whole biomarker-level distribution.

The results for these five selected biomarker-dementia endpoint associations are shown stratified for age, sex, obesity, diabetes, CVD, and *APOE* ε4 in Supplemental Tables 17-21. Generally, results were similar in subgroups defined by the first four factors. For *APOE* ε4, there was a consistent pattern towards stronger associations of inflammation biomarkers among *APOE* ε4-negative subjects. In line with this observation, the only statistically significant interaction found was between *APOE* ε4 polymorphism and the biomarker EN-RAGE for all-cause dementia (*p*=0.024, Supplemental Table 19).

The results of the sensitivity analyses are also shown in Supplemental Tables 17-21. When stratified by time of diagnosis, all selected biomarkers had a stronger association with dementia diagnoses occurring in the first 10 years of follow-up. However, significant associations were also observed for diagnoses in later years of follow-up. Besides, excluding subjects who died before their 80th birthday or had a sign of acute infection (CRP level >20mg/L) did not alter the results to any relevant extent. When those five biomarkers whose best fitting function was not the linear one were modelled with their best fitting polynomial, they showed stronger associations with the respective outcomes, but this mostly did not change the conclusions about their associations with the dementia outcomes (Supplemental Table 22). Exceptions were the association for Beta-NGF and all-cause dementia as well as the associations of CCL3, CCL20, and IL20-RA with vascular dementia, which became statistically significant with the best fitting function.

## Discussion

To our knowledge, this is the first prospective cohort study to analyse a whole panel of inflammation-related, blood-based biomarkers for all-cause dementia, Alzheimer’s disease, and vascular dementia incidence. We identified a high number of statistically significantly associated proteins with at least one of the outcomes, even after FDR correction. However, only a few biomarkers were strongly and independently associated with dementia outcomes because of a high inter-correlation between the biomarkers. The identified independent biomarkers include CX3CL1 (associated with all-cause dementia), EN-RAGE (associated with all-cause dementia and Alzheimer’s disease), LAP TGF-beta-1 (associated with Alzheimer’s disease), and VEGF-A (associated with vascular dementia). Each of these biomarkers is only one marker of an inflammatory protein cluster, in which the majority of biomarkers are associated with dementia.

### Previous studies examining a set of inflammatory biomarkers

A few previous studies, mostly with a cross-sectional study design, investigated the association between single inflammatory biomarkers and all-cause dementia or Alzheimer’s disease [6–9]. To our knowledge, only two previous studies examined a whole panel of inflammatory biomarkers for dementia as the outcome in a cross-sectional design. In the BioFINDER study, Whelan and colleagues [32] measured 270 proteins with the Olink immunoassay in cerebrospinal fluid (CSF) and plasma of 161 Alzheimer’s disease patients, 75 amyloid beta positive (Aβ+) patients with mild cognitive impairment (MCI+), and 415 amyloid beta negative (Aβ−) cognitively normal individuals (MCI−). Interestingly, approximately half of the CSF proteins correlated at least modestly with their analogues in plasma, indicating that findings in plasma samples partially reflected the situation in CSF. Compared to Aβ−/MCI− individuals, CSF levels of 32 proteins and plasma levels of 33 proteins were statistically significantly associated with Alzheimer’s disease (false discovery corrected *p*-value < 0.05). The comparison of Aβ+/MCI+ patients with Aβ−/MCI− individuals was replicated in an independent cohort. Thereby, 10 CSF and six plasma markers could be replicated. However, a replication analysis in an independent sample was not performed for Alzheimer’s disease. Six of the identified 33 proteins for Alzheimer’s disease in plasma samples corresponded with our findings in serum samples and can now be considered replicated by our study (Casp-8, CXCL5, CXCL6, ST1A1, TRAIL, uPA). Gaetani and colleagues measured biomarker levels of the Olink inflammation panel in CSF samples of 34 AD-MCI cases and 25 controls having other neurological diseases (OND) [33]. In univariate analyses, 11 of 46 analysed biomarkers were found to have the highest discriminatory ability between AD-MCI and OND. Four of those biomarkers (SIRT2, HGF, MMP-10, CXCL5) were also selected as discriminatory factors during penalized logistic regression (LASSO regression).

However, the studies of Whelan and Gaetani were cross-sectional, and evidence from longitudinal studies on this field is still sparse [10]. The recently published longitudinal study of Walker et al. [34] reported statistically significant associations between inflammatory biomarkers measured in midlife (C-reactive protein and a composite score of fibrinogen, white blood cell count, von Willebrand factor, and factor VIII) and cognitive decline over 20 years in a population-based cohort study with 12,336 participants. Our longitudinal results with a broad panel of inflammatory proteins and the endpoints all-cause dementia, Alzheimer’s disease, and vascular dementia complement and expand these findings.

### Independently associated biomarkers

#### CX3CL1

The biomarker CX3CL1, which is also commonly known as Fractalkine in humans, was independently associated with all-cause dementia and among the list of statistically significant biomarkers for Alzheimer’s disease and vascular dementia in our study. This biomarker is a chemokine binding to its receptor C-X3-C motif chemokine receptor 1 (CX3CR1) in a one-to-one relationship. While CX3CL1 is usually expressed in neurons, CX3CR1 is expressed on microglia. In the case of neuroinflammation, CX3CL1 regulates microglial activation by reducing the release of pro-inflammatory products [35]. Whether the effect of CX3CL1 is neuroprotective or neurotoxic in diseases like dementia is still controversially discussed in the literature. The current opinion is that this depends on the disease state, the affected CNS area, and the local concentration of the CX3CL1/CX3CR1 complex [35, 36].

Nevertheless, due to the regulatory function in inflammation, this biomarker is a promising therapeutic target. A recent Polish study reported on the predictive ability of CX3CL1 as a biomarker in the early development of mild cognitive impairment (MCI) and Alzheimer’s disease [37]. In this study, significantly higher CSF and blood levels of CX3CL1 were found in MCI and Alzheimer’s disease patients compared to cognitively healthy controls. We now confirm these results with longitudinal data, including 17 years of follow-up.

#### EN-RAGE

EN-RAGE was independently associated with all-cause dementia and Alzheimer’s disease. In addition, EN-RAGE was significantly associated with vascular dementia. EN-RAGE is also often referenced as S100-A12. The S100-protein family has already been shown multiple times to be related to Alzheimer’s disease [38]. However, the S100-A12 protein (EN-RAGE) is the least studied S100 protein in the context of Alzheimer’s disease and dementia [38]. In the only available study, Shepherd and colleagues [39] revealed associations of EN-RAGE with senile plaques, reactive glia, and neurons in brain samples of sporadic and familial (PS-1) Alzheimer’s disease cases in a cross-sectional study. Our study is the first longitudinal cohort study reporting on this association.

EN-RAGE is a calcium-, zinc-, and copper-binding protein. In previous studies, it was shown to be associated with diseases like heart failure [40] and coronary artery disease (CAD) in diabetes patients [41]. Recently, Feng and colleagues [42] reported significantly elevated EN-RAGE concentrations in patients with traumatic brain injury compared to controls. In this study, EN-RAGE showed great potential as a marker for ongoing inflammatory processes in the brain. RAGE, the receptor EN-RAGE binds to, is additionally known to be involved in inflammatory processes related to ageing and neurodegeneration [43, 44].

#### LAP-TGF-beta-1

LAP TGF-beta-1 is an anti-inflammatory cytokine that was independently associated with Alzheimer’s disease in our study (OR [95% CI]: 1.46 [1.21–1.76]). Additionally, it was significantly associated with all-cause dementia and vascular dementia. This biomarker consists of two components, latency-associated peptide (LAP) and transforming growth factor beta-1 (TGF-beta-1), which are non-covalently linked to each other in the intracellular environment. Thereby, LAP keeps TGF-beta-1 biologically inactive [45]. When activated, TGF-beta-1 binds to its receptor transforming growth factor-ß receptor type I (TßR-1), protecting neurons against Aß deposits and apoptosis [46, 47]. However, controversial findings have been reported on concentrations of this biomarker in Alzheimer’s disease [48]. The current theory is that the level of TGF-beta-1 in the body might depend on disease progression [48]. According to this theory, the reported elevated levels of TGF-beta-1 in our study might show an early response to commencing neurodegenerative processes in Alzheimer’s disease. A recent study additionally reported on the specificity of TGF-beta-1 for Alzheimer’s disease and vascular dementia compared to Parkinson’s disease dementia (PDD) [49].

#### VEGF-A

In our cohort, VEGF-A was independently associated with vascular dementia (OR [95% CI]: 1.43 [1.20–1.70]) and also significantly associated with all-cause dementia and Alzheimer’s disease. VEGF-A belongs to the vascular endothelial growth factor (VEGF) family and induces endothelial cell growth, cell migration, and permeabilization of blood vessels. Like other members of this family, VEGF-A induces the receptors VEGF receptor 1 and 2 (VEGFR-1 and VEGFR-2) [50]. In vascular dementia, VEGF-A is reported to be involved in microvessel loss and blood-brain barrier breakdown [51]. It was shown in the same study in mice that VEGF-A is involved in the hypoxia-inducible factor 1α-Lipocalin2-VEGFA (HIF-1α-LCN2-VEGFA) axis. Other groups have also shown an involvement of VEGF-A in increasing blood-brain barrier permeability [52, 53]. Hence, blocking VEGF-A signalling might be a promising therapeutic target [54].

### Inflammatory proteins prominently discussed in dementia research and biomarker clusters

Interestingly, the frequently discussed inflammatory biomarker IL-6 was not significantly associated with any dementia outcome in our study but highly correlated with EN-RAGE and VEGF-A [6–8]. However, apart from the inflammatory biomarkers discussed above, many others were statistically significantly associated with dementia outcomes as well but highly correlated with the highlighted proteins. IL-10, for example, is currently discussed by others as a risk factor for Alzheimer’s disease [9]. In our study, IL-10 was also statistically significantly associated with all-cause dementia and vascular dementia even after correction for multiple testing. In addition, a subunit of the IL-10 receptor (IL-10RB) was significantly associated with all-cause dementia and Alzheimer’s disease. Both IL-10 and IL-10RB were highly correlated with VEGF-A and IL-10RB, additionally with LAP TGF-beta-1 and CX3CL1. Due to the high correlation of these biomarkers, it is not possible to decide with our study design which of the biomarkers are the most clinically relevant ones and are causally associated with the outcome. Basic research is needed to elucidate this open question and the role of the identified biomarkers in the aetiology of dementia. The underlying mechanisms are likely to be complex because it is known that the multifactorial process of inflammation comes along with increases in the levels of many inflammatory proteins. Therefore, it might be necessary to look into inflammatory protein networks/clusters rather than focusing on single proteins in future studies. For example, for Alzheimer’s disease, we identified two such protein clusters. The EN-RAGE and the LAP TGF-beta-1 cluster consist of respectively nine inflammatory proteins significantly associated with Alzheimer’s disease (Supplemental Tables 14 and 15). The overlap of the two clusters is only three proteins (HGF, CD244, and uPA).

### Role of APOE ε4 polymorphism

*APOE* ε4-negative subjects had stronger associations between inflammation biomarkers and dementia outcomes than *APOE* ε4-positive individuals. Interestingly, the interaction of *APOE* ε4 and EN-RAGE for all-cause dementia was statistically significant. One explanation could be that the absolute Alzheimer’s disease risk of *APOE* ε4 carriers is so pronounced that the additional presence or absence of a weaker risk factor, such as inflammation, may not have much impact. In contrast, the potential impact of inflammation on the total dementia risk of *APOE* ε4 non-carriers is relatively high compared to other dementia risk factors.

### Clinical relevance of the findings

With a 41% increased dementia risk by 1 SD increase of either CX3CL1 or EN-RAGE levels, the associations of these two inflammatory biomarkers with dementia were stronger than the associations of male sex, education, CVD, and diabetes with all-cause dementia in our cohort. The strengths of the associations were comparable to the one of physical inactivity. Only age, APOE ε4 genotype, and a depression treated with anti-depressants (likely as an indicator of a major depression) were stronger risk factors for all-cause dementia in our study. This highlights the relevance of chronic inflammation as a strong, independent factor associated with dementia.

The identified inflammatory biomarkers should be further studied in basic science to further elucidate mechanisms and how they contribute to dementia pathogenesis. After this has been done, they may be used as drug targets, early diagnostic markers, and components of dementia prediction scores. Our results also add to current discussions about the potential of the anti-inflammatory drug low-dose aspirin in dementia prevention. A recent analysis of two cohort studies showed that the use of low-dose aspirin for 10 years and more is associated with a lower risk for all-cause dementia, Alzheimer’s disease, and vascular dementia in patients with pre-existing coronary heart disease [55].

### Strengths and limitations

The strengths of this study comprise a large sample size, the representative sample of an older adult population, a long follow-up period (17 years), and the prospective cohort design limiting the risk of reverse causality. Moreover, the diversity of inflammatory biomarkers (72 biomarkers analysed) and the high sensitivity and specificity of Olink’s proximity extension assays [19, 20] used for the biomarker measurements can be assigned to the study’s strengths.

The observational study design is one of the limitations of this study. Although analyses were controlled for confounders, residual confounding cannot be entirely excluded. Apart from this, the latency between the onset and the clinical diagnosis of dementia can be longer than the follow-up time of 17 years [56]. However, results were still statistically significant after excluding events in the first 10 years of follow-up. Thus, we can assume that there is no strong indication of reverse causality in our study results.

In the ESTHER study, dementia information is collected via GPs. After a referral to various neurologists, psychiatrists, memory clinics, or other specialized providers in the study region, diagnoses were obtained from the medical records of specialists. Although this process reflects the community-based clinical setting in Germany and enhances the generalizability of the study, it implicates a possible occurrence of underdiagnosed dementia. In addition, dementia diagnostics were performed heterogeneously in routine practice (not following any study protocol) and dementia subtypes were often not differentially assessed. This may be one reason why the ratio of Alzheimer’s disease to vascular dementia diagnoses is not as high in our study as in other studies with homogenous subtype diagnostics based on biomarkers of Alzheimer’s disease pathology among all study participants.

Due to cost issues, biomarker measurements were performed in a case-cohort design and not in the total cohort and only in baseline samples and not additionally in follow-up samples. The latter point might have led to an underestimation of the results because inflammation status could change during the follow-up. Furthermore, cost issues did not allow replication of the results in another independent study yet and this should be aimed for by future research. Such an external validation is crucial in studies with omics data to increase the generalizability of the results.

The biomarkers TNF and IFN-gamma had to be excluded since the proportion of values below LOD was >25% in the total study sample. After Olink improved the inflammation panel in 2019, better results could be achieved for these biomarkers. However, the improved panel was only used in a fraction of our study sample (*n* = 388). When analysing the data only in this sub-sample, TNF and IFN-gamma showed weak and not statistically significant associations with all three dementia outcomes (Supplemental Table 23).

Lastly, it has to be stated that our study results refer to an almost exclusively Caucasian population with blood samples taken between the ages of 50 and 75 years and may not be generalized to other types of populations.

## Conclusion

This study showed that 58 out of 72 tested proteins of the inflammatory proteome in blood were significantly associated with all-cause dementia incidence even after correction for multiple testing. Several inflammatory proteins were further associated with Alzheimer’s disease and vascular dementia. The biomarkers CX3CL1, EN-RAGE, LAP TGF-beta-1, and VEGF-A had strong and independent associations with dementia outcomes and may have great potential as drug targets, early diagnostic markers, and components of dementia prediction scores. However, due to the observed high inter-correlation of inflammatory biomarkers, it should be noted that not only single biomarkers but also clusters of increased inflammatory protein levels may play a role in dementia pathogenesis or risk prediction. The complex interrelationships in these clusters are not yet understood and require further research.

## Supplementary Information

**Additional file 1: Supplemental Table 1.** List of biomarkers measured with Olink Proseek® Multiplex Inflammation I96x96 kits. **Supplemental Table 2.** Baseline characteristics of participants with (*n*=6284) and without (*n*=3656) available dementia information and blood samples. **Supplemental Table 3.** Baseline characteristics of selected (*n*=1278) and non-selected (*n*=4456) controls. **Supplemental Table 4.** Comparison of median Olink Inflammation panel biomarker levels between all-cause dementia cases (*n*=504) and controls (*n*=1278). **Supplemental Table 5.** Comparison of median Olink Inflammation panel biomarker levels between Alzheimer’s disease cases (*n*=163) and controls (*n*=1278). **Supplemental Table 6.** Comparison of median Olink Inflammation panel biomarker levels between vascular dementia cases (*n*=195) and controls (*n*=1278). **Supplemental Table 7.** Non-significant associations of Olink Inflammation panel biomarker levels with all-cause dementia incidence (FDR ≥ 0.05). **Supplemental Table 8.** Non-significant associations of Olink Inflammation panel biomarker levels with Alzheimer’s disease incidence (FDR ≥ 0.05). **Supplemental Table 9.** Non-significant associations of Olink Inflammation panel biomarker levels with vascular dementia incidence (FDR ≥ 0.05). **Supplemental Table 10.** Replication of strong associations of Olink Inflammation panel biomarker levels with all-cause dementia incidence with multivariate, weighted Cox proportional hazards regression. **Supplemental Table 11.** Replication of strong associations of Olink Inflammation panel biomarker levels with Alzheimer’s disease incidence with multivariate, weighted Cox proportional hazards regression. **Supplemental Table 12.** Replication of strong associations of Olink Inflammation panel biomarker levels with vascular dementia incidence with multivariate, weighted Cox proportional hazards regression. **Supplemental Table 13.** Olink Inflammation panel biomarkers in the CX3CL1 cluster. **Supplemental Table 14.** Olink Inflammation panel biomarkers in the EN-RAGE cluster. **Supplemental Table 15.** Olink Inflammation panel biomarkers in the LAP TGF-beta-1 cluster. **Supplemental Table 16.** Olink Inflammation panel biomarkers in the VEGF-A cluster. **Supplemental Table 17.** Exploratory subgroup and sensitivity analyses for the association of CX3CL1 and all-cause dementia. **Supplemental Table 18.** Exploratory subgroup and sensitivity analyses for the association of EN-RAGE and all-cause dementia. **Supplemental Table 19.** Exploratory subgroup and sensitivity analyses for the association of EN-RAGE and Alzheimer’s disease. **Supplemental Table 20.** Exploratory subgroup and sensitivity analyses for the association of LAP TGF-beta-1 and Alzheimer’s disease. **Supplemental Table 21.** Exploratory subgroup and sensitivity analyses for the association of VEGF-A and vascular dementia. **Supplemental Table 22.** Associations of five Olink inflammation panel biomarker levels with all-cause dementia, Alzheimer’s disease, and vascular dementia incidence modelled linearly and modelled with their best fitting function. **Supplemental Table 23.** Associations of IFN-gamma and TNF levels with all-cause dementia, Alzheimer’s disease and vascular dementia incidence for participants included in the third wave of measurements (t3) (*n*=388).

## Data Availability

Data are available on request.

## Abbreviations

AD: Alzheimer’s disease
APOE: Apolipoprotein E
Aβ-: Amyloid beta negative
Aβ+: Amyloid beta positive
BMI: Body mass index
CAD: Coronary artery disease
CHD: Coronary heart disease
CNS: Central nervous system
CRP: C-reactive protein
CSF: Cerebrospinal fluid
CVD: Cardiovascular disease
CX3CL1: Fractalkine
CX3CR1: C-X3-C motif chemokine receptor 1
EN-RAGE: S100-A12
ESTHER: Epidemiologische Studie zu Chancen der Verhütung, Früherkennung und optimierten Therapie chronischer Erkrankungen in der älteren Bevölkerung [German]
FDR: False discovery rate
GPs: General practitioners
HIF-1α-LCN2-VEGFA: Hypoxia-inducible factor 1α-Lipocalin2-VEGFA
IL-1ß: Interleukin-1ß
IL-6: Interleukin-6
IP-10: Interferon-γ-inducible protein 10
IQR: Interquartile range
LAP TGF-beta-1: Latency-associated peptide transforming growth factor beta-1
LOD: Limit of detection
MCI-: Cognitively normal individuals
MCI+: Mild cognitive impairment
MCMC: Markov Chain Monte Carlo
MCP-1: Monocyte protein-1
NPX: Normalized Protein eXpression
OND: Other neurological diseases
ORs: Odds ratios
PCR: Polymerase chain reaction
PDD: Parkinson’s disease dementia
PEA: Proximity extension assay
qPCR: Quantitative polymerase chain reaction
SD: Standard deviation
SNP: Single-nucleotide polymorphism
TNF-α: Tumour necrosis factor α
TßR-1: Transforming growth factor-ß receptor type I
VD: Vascular dementia
VEGF: Vascular endothelial growth factor
VEGF-A: Vascular endothelial growth factor A
VEGFR-1: Vascular endothelial growth factor receptor 1
α1-AT: α1-Antichymotrypsin
