# Supplementary material for: Association of the inflammation-related proteome with dementia development at older age: results from a large, prospective, population-based cohort study
Source: Alzheimers Res Ther. 2022 Sep 9;14:128. doi: 10.1186/s13195-022-01063-y (PMC9461133; doi:10.1186/s13195-022-01063-y)
Supplement: Supplementary file 1 — Additional file 1: Supplemental Table 1. List of biomarkers measured with Olink Proseek® Multiplex Inflammation I96x96 kits. Supplemental Table 2. Baseline characteristics of participants with (n=6284) and without (n=3656) available dementia information and blood samples. Supplemental Table 3. Baseline characteristics of selected (n=1278) and non-selected (n=4456) controls. Supplemental Table 4. Comparison of median Olink Inflammation panel biomarker levels between all-cause dementia cases (n=504) and controls (n=1278). Supplemental Table 5. Comparison of median Olink Inflammation panel biomarker levels between Alzheimer’s disease cases (n=163) and controls (n=1278). Supplemental Table 6. Comparison of median Olink Inflammation panel biomarker levels between vascular dementia cases (n=195) and controls (n=1278). Supplemental Table 7. Non-significant associations of Olink Inflammation panel biomarker levels with all-cause dementia incidence (FDR ≥ 0.05). Supplemental Table 8. Non-significant associations of Olink Inflammation panel biomarker levels with Alzheimer’s disease incidence (FDR ≥ 0.05). Supplemental Table 9. Non-significant associations of Olink Inflammation panel biomarker levels with vascular dementia incidence (FDR ≥ 0.05). Supplemental Table 10. Replication of strong associations of Olink Inflammation panel biomarker levels with all-cause dementia incidence with multivariate, weighted Cox proportional hazards regression. Supplemental Table 11. Replication of strong associations of Olink Inflammation panel biomarker levels with Alzheimer’s disease incidence with multivariate, weighted Cox proportional hazards regression. Supplemental Table 12. Replication of strong associations of Olink Inflammation panel biomarker levels with vascular dementia incidence with multivariate, weighted Cox proportional hazards regression. Supplemental Table 13. Olink Inflammation panel biomarkers in the CX3CL1 cluster. Supplemental Table 14. Olink Inflammation panel biomarke [file 13195_2022_1063_MOESM1_ESM.docx]

**Supplemental Material to Article**

“Association of the inflammation-related proteome with dementia development at older age: results from a large, prospective, population-based cohort study”

Table of Contents

[**Supplemental Table 1.** List of biomarkers measured with Olink Proseek® Multiplex Inflammation I96x96 kits. 3](#_Toc109901051)

[**Supplemental Table 2.** Baseline characteristics of participants with (n=6284) and without (n=3656) available dementia information and blood samples. 6](#_Toc109901052)

[**Supplemental Table 3.** Baseline characteristics of selected (n=1278) and non-selected (n=4456) controls 7](#_Toc109901053)

[**Supplemental Table 4.** Comparison of median Olink Inflammation panel biomarker levels between all-cause dementia cases (n=504) and controls (n=1278) 8](#_Toc109901054)

[**Supplemental Table 5.** Comparison of median Olink Inflammation panel biomarker levels between Alzheimer’s disease cases (n=163) and controls (n=1278) 10](#_Toc109901055)

[**Supplemental Table 6.** Comparison of median Olink Inflammation panel biomarker levels between vascular dementia cases (n=195) and controls (n=1278) 12](#_Toc109901056)

[**Supplemental Table 7.** Non-significant associations of Olink Inflammation panel biomarker levels with all-cause dementia incidence (FDR ≥ 0.05). 14](#_Toc109901057)

[**Supplemental Table 8.** Non-significant associations of Olink Inflammation panel biomarker levels with Alzheimer’s disease incidence (FDR ≥ 0.05) 15](#_Toc109901058)

[**Supplemental Table 9.** Non-significant associations of Olink Inflammation panel biomarker levels with vascular dementia incidence (FDR ≥ 0.05) 17](#_Toc109901059)

[**Supplemental Table 10.** Replication of strong associations of Olink Inflammation panel biomarker levels with all-cause dementia incidence with multivariate, weighted Cox proportional hazards regression. 18](#_Toc109901060)

[**Supplemental Table 11.** Replication of strong associations of Olink Inflammation panel biomarker levels with Alzheimer’s disease incidence with multivariate, weighted Cox proportional hazards regression. 19](#_Toc109901061)

[**Supplemental Table 12.** Replication of strong associations of Olink Inflammation panel biomarker levels with vascular dementia incidence with multivariate, weighted Cox proportional hazards regression. 20](#_Toc109901062)

[**Supplemental Table 13.** Olink Inflammation panel biomarkers in the CX3CL1 cluster 21](#_Toc109901063)

[**Supplemental Table 14.** Olink Inflammation panel biomarkers in the EN-RAGE cluster 22](#_Toc109901064)

[**Supplemental Table 15.** Olink Inflammation panel biomarkers in the LAP TGF-beta-1 cluster 23](#_Toc109901065)

[**Supplemental Table 16.** Olink Inflammation panel biomarkers in the VEGF-A cluster 24](#_Toc109901066)

[**Supplemental Table 17.** Exploratory subgroup and sensitivity analyses for the association of CX3CL1 and all-cause dementia 25](#_Toc109901067)

[**Supplemental Table 18.** Exploratory subgroup and sensitivity analyses for the association of EN-RAGE and all-cause dementia 26](#_Toc109901068)

[**Supplemental Table 19.** Exploratory subgroup and sensitivity analyses for the association of EN-RAGE and Alzheimer’s disease 27](#_Toc109901069)

[**Supplemental Table 20.** Exploratory subgroup and sensitivity analyses for the association of LAP TGF-beta-1 and Alzheimer’s disease 28](#_Toc109901070)

[**Supplemental Table 21.** Exploratory subgroup and sensitivity analyses for the association of VEGF-A and vascular dementia 29](#_Toc109901071)

[**Supplemental Table 22.** Associations of five Olink inflammation panel biomarker levels with all-cause dementia, Alzheimer’s disease, and vascular dementia incidence modelled linearly and modelled with their best fitting function. 30](#_Toc109901072)

[**Supplemental Table 23.** Associations of IFN-gamma and TNF levels with all-cause dementia, Alzheimer’s disease and vascular dementia incidence for participants included in the third wave of measurements (t3) (n=388). 31](#_Toc109901073)

Supplemental Table 1. List of biomarkers measured with Olink Proseek® Multiplex Inflammation I96x96 kits.

| **Abbreviation** | **Biomarker name** | **Values < LOD** |
| --- | --- | --- |
| 4E-BP1 | Eukaryotic translation initiation factor 4E-binding protein 1 | 0% |
| ADA | Adenosine Deaminase | 0% |
| ARTN | Artemin | 76% |
| AXIN1 | Axin-1 | 5% |
| Beta-NGF | Beta-nerve growth factor | 12% |
| CASP-8 | Caspase-8 | 0% |
| CCL11 | Eotaxin | 0% |
| CCL19 | C-C motif chemokine 19 | 0% |
| CCL20 | C-C motif chemokine 20 | 0% |
| CCL23 | C-C motif chemokine 23 | 0% |
| CCL25 | C-C motif chemokine 25 | 0% |
| CCL28 | C-C motif chemokine 28 | 2% |
| CCL3 | C-C motif chemokine 3 | 0% |
| CCL4 | C-C motif chemokine 4 | 0% |
| CD244 | Natural killer cell receptor 2B4 | 0% |
| CD40 | CD40L receptor | 0% |
| CD5 | T-cell surface glycoprotein CD5 | 0% |
| CD6 | T cell surface glycoprotein CD6 isoform | 0% |
| CD8A | T-cell surface glycoprotein CD8 alpha chain | 0% |
| CDCP1 | CUB domain-containing protein 1 | 0% |
| CSF-1 | Macrophage colony-stimulating factor 1 | 0% |
| CST5 | Cystatin D | 3% |
| CX3CL1 | Fractalkine | 0% |
| CXCL1 | C-X-C motif chemokine 1 | 0% |
| CXCL10 | C-X-C motif chemokine 10 | 0% |
| CXCL11 | C-X-C motif chemokine 11 | 0% |
| CXCL5 | C-X-C motif chemokine 5 | 0% |
| CXCL6 | C-X-C motif chemokine 6 | 0% |
| CXCL9 | C-X-C motif chemokine 9 | 0% |
| DNER | Delta and Notch-like epidermal growth factor-related receptor | 0% |
| EN-RAGE | Protein S100-A12 | 0% |
| FGF-19 | Fibroblast growth factor 19 | 0% |
| FGF-21 | Fibroblast growth factor 21 | 0% |
| FGF-23 | Fibroblast growth factor 23 | 25% |
| FGF-5 | Fibroblast growth factor 5 | 40% |
| Flt3L | Fms-related tyrosine kinase 3 ligand | 0% |
| GDNF | Glial cell line-derived neurotrophic factor | 25% |
| HGF | Hepatocyte growth factor | 0% |
| IFN_gamma | Interferon gamma | 81% |
| IL1_alpha | Interleukin-1 alpha | 85% |
| IL-10 | Interleukin-10 | 3% |
| IL-10RA | Interleukin-10 receptor subunit alpha | 22% |
| IL-10RB | Interleukin-10 receptor subunit beta | 0% |
| IL-12B | Interleukin-12 subunit beta | 0% |
| IL13 | Interleukin-13 | 86% |
| IL-15RA | Interleukin-15 receptor subunit alpha | 20% |
| IL-17A | Interleukin-17A | 28% |
| IL-17C | Interleukin-17C | 32% |
| IL-18 | Interleukin-18 | 0% |
| IL-18R1 | Interleukin-18 receptor 1 | 0% |
| IL2 | Interleukin-2 | 98% |
| IL20 | Interleukin-20 | 78% |
| IL-20RA | Interleukin-20 receptor subunit alpha | 47% |
| IL22-RA1 | Interleukin-22 receptor subunit alpha-1 | 83% |
| IL24 | Interleukin-24 | 90% |
| IL2RB | Interleukin-2 receptor subunit beta | 66% |
| IL33 | Interleukin-33 | 97% |
| IL4 | Interleukin-4 | 85% |
| IL-5 | Interleukin-5 | 62% |
| IL-6 | Interleukin-6 | 1% |
| IL-7 | Interleukin-7 | 0% |
| IL-8 | Interleukin-8 | 0% |
| LAP TGF-beta-1 | Latency-associated peptide transforming growth factor beta-1 | 0% |
| LIF | Leukemia inhibitory factor | 65% |
| LIFR | Leukemia inhibitory factor receptor | 0% |
| MCP-1 | Monocyte chemotactic protein 1 | 0% |
| MCP-2 | Monocyte chemotactic protein 2 | 0% |
| MCP-3 | Monocyte chemotactic protein 3 | 5% |
| MCP-4 | Monocyte chemotactic protein 4 | 0% |
| MMP-1 | Matrix metalloproteinase-1 | 0% |
| MMP-10 | Matrix metalloproteinase-10 | 0% |
| NRTN | Neurturin | 72% |
| NT-3 | Neurotrophin-3 | 13% |
| OPG | Osteoprotegerin | 0% |
| OSM | Oncostatin-M | 0% |
| PD-L1 | Programmed cell death 1 ligand 1 | 0% |
| SCF | Stem cell factor | 0% |
| SIRT2 | SIR2-like protein 2 | 7% |
| SLAMF1 | Signaling lymphocytic activation molecule | 9% |
| ST1A1 | Sulfotransferase 1A1 | 4% |
| STAMBP | STAM-binding protein | 0% |
| TGF-alpha | Transforming growth factor alpha | 0% |
| TNF | Tumor necrosis factor | 70% |
| TNFB | TNF-beta | 0% |
| TNFRSF9 | Tumor necrosis factor receptor superfamily member 9 | 0% |
| TNFSF14 | Tumor necrosis factor ligand superfamily member 14 | 0% |
| TRAIL | TNF-related apoptosis-inducing ligand | 0% |
| TRANCE | TNF-related activation-induced cytokine | 0% |
| TSLP | Thymic stromal lymphopoietin | 87% |
| TWEAK | Tumor necrosis factor (Ligand) superfamily, member 12 | 0% |
| uPA | Urokinase-type plasminogen activator | 0% |
| VEGF-A | Vascular endothelial growth factor-A | 0% |

Note: Grey shade indicates biomarkers with 25% or more of the values below the lower limit of detection (LOD)

Supplemental Table 2. Baseline characteristics of participants with (n=6284) and without (n=3656) available dementia information and blood samples.

| **Baseline characteristics** | **Participants with dementia information**  **(n=6284)** | **Participants without dementia information**  **(n=3656)^a^** |
| --- | --- | --- |
| **Age (years)** |  |  |
| 50-64 | 4014 (63.9) | 2090 (57.2) |
| 65-69 | 1405 (22.4) | 870 (23.8) |
| 70-75 | 865 (13.8) | 696 (19.0) |
| **Sex** |  |  |
| Female | 3412 (54.3) | 2050 (56.1) |
| Male | 2872 (45.7) | 1606 (43.9) |
| **Education (years)** |  |  |
| ≤ 9 | 4511 (73.5) | 2724 (76.7) |
| 10-11 | 894 (14.6) | 478 (13.5) |
| ≥ 12 | 733 (11.9) | 348 (9.8) |
| **Physical activity** |  |  |
| Inactive | 1206 (19.2) | 913 (25.1) |
| Low | 2843 (45.4) | 1683 (46.2) |
| Medium or high | 2220 (35.4) | 1045 (28.7) |
| **BMI (kg/m²)** |  |  |
| < 25 | 1744 (27.8) | 980 (26.8) |
| 25-<30 | 2957 (47.1) | 1718 (47.1) |
| ≥30 | 1572 (25.1) | 953 (26.1) |
| **Cardiovascular disease** |  |  |
| No | 5064 (80.6) | 2884 (79.0) |
| Yes | 1219 (19.4) | 769 (21.1) |
| **Diabetes** |  |  |
| No | 5307 (85.6) | 2923 (83.8) |
| Yes | 891 (14.4) | 564 (16.2) |
| **Life-time history  of depression** |  |  |
| No | 5357 (85.4) | 3138 (86.3) |
| Yes, without current  pharmacotherapy | 718 (11.5) | 393 (10.8) |
| Yes, with current  pharmacotherapy | 198 (3.2) | 104 (2.9) |
| **APOE genotypes** |  |  |
| ԑ2/ԑ2 | 42 (0.7) | 29 (0.9) |
| ԑ2/ԑ3 | 835 (14.7) | 452 (14.5) |
| ԑ2/ԑ4 | 205 (3.6) | 107 (3.4) |
| ԑ3/ԑ3 | 3324 (58.5) | 1821 (58.4) |
| ԑ3/ԑ4 | 1197 (21.1) | 636 (20.4) |
| ԑ4/ԑ4 | 84 (1.5) | 71 (2.3) |

Abbreviations: CI, confidence interval; BMI, body mass index; *APOE*, apolipoprotein E;

^a^The sample additionally includes n=73 participants with missing blood samples, which were also not available for the analyses (cf. flowchart in Figure 1).

Supplemental Table 3. Baseline characteristics of selected (n=1278) and non-selected (n=4456) controls

| **Baseline characteristics** | **Non-selected controls**  **(n=4456)** | **Selected controls**  **(n=1278)** |
| --- | --- | --- |
| **Age (years)** |  |  |
| 50-64 | 3028 (67.9) | 802 (62.8) |
| 65-69 | 941 (21.1) | 301 (23.6) |
| 70-75 | 489 (11.0) | 175 (13.7) |
| **Sex** |  |  |
| Female | 2426 (54.4) | 703 (55.0) |
| Male | 2032 (45.6) | 575 (45.0)) |
| **Education (years)** |  |  |
| ≤ 9 | 3138 (71.8) | 953 (76.1) |
| 10-11 | 673 (15.4) | 168 (13.4) |
| ≥ 12 | 549 (12.6) | 131 (10.5) |
| **Physical activity*** |  |  |
| Inactive | 820 (18.4) | 233 (18.3) |
| Low | 2001 (45.0) | 594 (46.6) |
| Medium or high | 1626 (36.6) | 448 (35.1) |
| **BMI (kg/m²)** |  |  |
| < 25 | 1251 (28.1) | 334 (26.2) |
| 25-<30 | 2107 (47.3) | 604 (47.5) |
| ≥30 | 1095 (24.6) | 335 (26.3) |
| **Cardiovascular disease** |  |  |
| No | 3649 (81.9) | 1023 (80.1) |
| Yes | 808 (18.1) | 255 (19.9) |
| **Diabetes** |  |  |
| No | 3793 (86.2) | 1083 (85.8) |
| Yes | 607 (13.8) | 180 (14.3) |
| **Life-time history  of depression** |  |  |
| No | 3785 (85.1) | 1100 (86.1) |
| Yes, without current  pharmacotherapy | 533 (12.0) | 137 (10.7) |
| Yes, with current  pharmacotherapy | 129 (2.9) | 41 (3.2) |
| **APOE genotypes** |  |  |
| ԑ2/ԑ2 | 24 (0.6) | 17 (1.4) |
| ԑ2/ԑ3 | 592 (14.8) | 181 (15.1) |
| ԑ2/ԑ4 | 145 (3.6) | 33 (2.8) |
| ԑ3/ԑ3 | 2371 (59.3) | 711 (59.5) |
| ԑ3/ԑ4 | 812 (20.3) | 244 (20.4) |
| ԑ4/ԑ4 | 52 (1.3) | 10 (0.8) |

Abbreviations: CI, confidence interval; BMI, body mass index; *APOE*, apolipoprotein E;

Supplemental Table 4. Comparison of median Olink Inflammation panel biomarker levels between all-cause dementia cases (n=504) and controls (n=1278)

| **Olink Biomarker** | **All-cause dementia cases (n=504)** | **Controls (n=1278)** | **p-value^a^** | **FDR^b^** |
| --- | --- | --- | --- | --- |
|  | **Median (IQR)** | **Median (IQR)** |  |  |
| ADA | 6.47 (6.09-6.82) | 6.38 (6.02-6.73) | **0.0041** | **0.0029** |
| AXIN1 | 3.72 (2.61-4.35) | 3.60 (2.49-4.26) | 0.0662 | 0.0536 |
| Beta-NGF | 0.61 (0.60-0.67) | 0.61 (0.43-0.77) | 0.3080 | 0.2807 |
| CASP-8 | 6.09 (5.15-7.09) | 5.93 (5.02-6.85) | **0.0248** | **0.0197** |
| CCL3 | 7.25 (6.65-8.00) | 7.03 (6.49-7.77) | **<0.0001** | **<0.0001** |
| CCL4 | 7.41 (6.87-8.10) | 7.25 (6.71-7.87) | **0.0006** | **0.0004** |
| CCL11 | 8.67 (8.27-9.06) | 8.54 (8.15-8.89) | **<0.0001** | **<0.0001** |
| CCL19 | 9.12 (8.49-9.84) | 8.84 (8.31-9.54) | **<0.0001** | **<0.0001** |
| CCL20 | 6.74 (6.04-7.57) | 6.44 (5.76-7.27) | **<0.0001** | **<0.0001** |
| CCL23 | 10.00 (9.62-10.41) | 9.86 (9.46-10.21) | **<0.0001** | **<0.0001** |
| CCL25 | 6.61 (6.16-7.01) | 6.39 (5.94-6.83) | **<0.0001** | **<0.0001** |
| CCL28 | 2.81 (2.53-3.15) | 2.70 (2.42-3.00) | **<0.0001** | **<0.0001** |
| CD5 | 5.99 (5.66-6.27) | 5.84 (5.54-6.14) | **<0.0001** | **<0.0001** |
| CD6 | 7.42 (6.99-7.83) | 7.34 (6.83-7.73) | **0.0038** | **0.0026** |
| CD40 | 12.61 (12.12-13.01) | 12.44 (11.98-12.89) | **0.0002** | **0.0001** |
| CD244 | 6.75 (6.44-7.07) | 6.63 (6.34-6.89) | **<0.0001** | **<0.0001** |
| CD8A | 9.47 (8.86-10.07) | 9.33 (8.73-9.91) | **0.0016** | **0.0010** |
| CDCP1 | 3.97 (3.45-4.49) | 3.64 (3.15-4.14) | **<0.0001** | **<0.0001** |
| CSF-1 | 11.01 (10.82-11.19) | 10.93 (10.73-11.12) | **<0.0001** | **<0.0001** |
| CST5 | 7.30 (6.94-7.67) | 7.15 (6.81-7.50) | **<0.0001** | **<0.0001** |
| CX3CL1 | 4.72 (4.46-5.06) | 4.57 (4.20-4.88) | **<0.0001** | **<0.0001** |
| CXCL1 | 10.39 (9.94-10.88) | 10.31 (9.84-10.74) | **0.0091** | **0.0066** |
| CXCL5 | 12.64 (12.00-13.20) | 12.44 (11.75-13.03) | **0.0002** | **0.0001** |
| CXCL6 | 9.39 (8.94-9.89) | 9.24 (8.73-9.70) | **<0.0001** | **<0.0001** |
| CXCL9 | 7.31 (6.87-7.89) | 6.98 (6.49-7.54) | **<0.0001** | **<0.0001** |
| CXCL10 | 9.96 (9.46-10.54) | 9.72 (9.26-10.24) | **<0.0001** | **<0.0001** |
| CXCL11 | 8.33 (7.63-8.92) | 8.06 (7.46-8.69) | **<0.0001** | **<0.0001** |
| DNER | 9.65 (9.40-9.86) | 9.56 (9.32-9.77) | **<0.0001** | **<0.0001** |
| EN-RAGE | 7.27 (6.46-8.18) | 7.00 (6.01-7.77) | **<0.0001** | **<0.0001** |
| FGF-19 | 8.82 (8.04-9.49) | 8.53 (7.82-9.24) | **<0.0001** | **<0.0001** |
| FGF-21 | 5.92 (5.05-6.70) | 5.65 (4.82-6.51) | **0.0008** | **0.0005** |
| FGF-23 | 0.93 (0.84-1.09) | 0.93 (0.64-1.15) | 0.3844 | 0.3552 |
| Flt3L | 9.77 (9.45-10.13) | 9.65 (9.33-9.98) | **<0.0001** | **<0.0001** |
| GDNF | 1.44 (1.29-2.04) | 1.43 (1.29-1.90) | **<0.0001** | **<0.0001** |
| HGF | 9.93 (9.45-10.28) | 9.73 (9.29-10.10) | **<0.0001** | **<0.0001** |
| IL-6 | 2.52 (1.98-3.43) | 2.30 (1.71-3.17) | **<0.0001** | **<0.0001** |
| IL-7 | 3.16 (2.65-3.73) | 3.09 (2.53-3.63) | **0.0169** | **0.0132** |
| IL-8 | 9.40 (7.74-11.96) | 9.19 (7.56-11.68) | 0.2557 | 0.2266 |
| IL-10 | 3.49 (3.09-3.88) | 3.36 (3.00-3.76) | **0.0002** | **0.0001** |
| IL-18 | 9.15 (8.68-9.54) | 8.90 (8.48-9.37) | **<0.0001** | **<0.0001** |
| IL-10RA | 0.71 (0.48-1.09) | 0.66 (0.43-0.98) | **0.0079** | **0.0057** |
| IL-10RB | 6.75 (6.50-7.01) | 6.64 (6.34-6.87) | **<0.0001** | **<0.0001** |
| IL-15RA | 1.51 (1.36-1.74) | 1.38 (1.28-1.67) | **<0.0001** | **<0.0001** |
| IL-18R1 | 9.17 (8.84-9.55) | 9.07 (8.68-9.40) | **<0.0001** | **<0.0001** |
| LAP TGF-beta-1 | 8.22 (7.89-8.55) | 8.07 (7.73-8.39) | **<0.0001** | **<0.0001** |
| LIF-R | 4.83 (4.59-5.09) | 4.71 (4.48-4.92) | **<0.0001** | **<0.0001** |
| MCP-1 | 12.52 (12.05-13.22) | 12.45 (12.00-12.98) | **0.0131** | **0.0098** |
| MCP-2 | 9.86 (9.34-10.24) | 9.69 (9.22-10.09) | **0.0001** | **0.0001** |
| MCP-3 | 3.89 (2.63-6.38) | 3.54 (2.46-6.13) | 0.0955 | 0.0810 |
| MCP-4 | 15.16 (14.69-15.65) | 15.04 (14.51-15.49) | **0.0002** | **0.0001** |
| MMP-1 | 15.90 (15.32-16.37) | 15.91 (15.18-16.43) | 0.6484 | 0.6319 |
| MMP-10 | 9.91 (9.48-10.30) | 9.75 (9.31-10.17) | **<0.0001** | **<0.0001** |
| NT-3 | 1.37 (1.14-1.65) | 1.31 (1.04-1.56) | **0.0006** | **0.0004** |
| OPG | 10.48 (10.16-10.79) | 10.26 (9.94-10.56) | **<0.0001** | **<0.0001** |
| OSM | 7.85 (6.97-8.99) | 7.87 (6.97-8.91) | 0.8246 | 0.8246 |
| PD-L1 | 6.26 (5.97-6.54) | 6.10 (5.82-6.38) | **<0.0001** | **<0.0001** |
| SCF | 9.69 (9.33-9.98) | 9.59 (9.24-9.86) | **<0.0001** | **<0.0001** |
| SIRT2 | 4.51 (3.61-5.17) | 4.38 (3.49-5.10) | 0.0794 | 0.0653 |
| SLAMF1 | 2.94 (2.66-3.34) | 2.87 (2.47-3.21) | **0.0002** | **0.0001** |
| ST1A1 | 4.20 (2.84-4.92) | 4.02 (2.87-4.81) | 0.0955 | 0.0801 |
| STAMBP | 5.77 (5.09-6.21) | 5.65 (5.05-6.11) | **0.0079** | **0.0057** |
| TGF-alpha | 4.91 (4.42-5.41) | 4.83 (4.30-5.30) | **0.0164** | **0.0126** |
| TNFB | 5.35 (5.03-5.65) | 5.35 (4.98-5.66) | 0.6034 | 0.5805 |
| TNFRSF9 | 7.61 (7.30-7.96) | 7.44 (7.11-7.78) | **<0.0001** | **<0.0001** |
| TNFSF14 | 7.86 (6.97-8.46) | 7.71 (6.94-8.33) | **0.0161** | **0.0122** |
| TRAIL | 8.40 (8.14-8.66) | 8.33 (8.05-8.57) | **<0.0001** | **<0.0001** |
| TRANCE | 5.29 (4.85-5.77) | 5.31 (4.80-5.75) | 0.5578 | 0.5296 |
| TWEAK | 9.27 (8.93-9.53) | 9.16 (8.87-9.43) | **<0.0001** | **<0.0001** |
| VEGF-A | 12.43 (11.88-12.89) | 12.17 (11.69-12.63) | **<0.0001** | **<0.0001** |
| 4E-BP1 | 8.03 (7.23-8.75) | 7.83 (6.90-8.60) | **0.0034** | **0.0023** |
| uPA | 9.94 (9.68-10.27) | 9.84 (9.53-10.11) | **<0.0001** | **<0.0001** |

Abbreviations: IQR, interquartile range; FDR, false discovery rate; For biomarker abbreviations, see Supplemental Table 1

NOTE: Numbers printed in bold are statistically significant (p < 0.05).

^a^Results of a Wilcoxon rank-sum test.

^b^P-values corrected for multiple testing by Benjamini and Hochberg method.

Supplemental Table 5. Comparison of median Olink Inflammation panel biomarker levels between Alzheimer’s disease cases (n=163) and controls (n=1278)

| **Olink Biomarker** | **Alzheimer’s disease (n=163)** | **Controls (n=1278)** | **p-value^a^** | **FDR^b^** |
| --- | --- | --- | --- | --- |
|  | **Median (IQR)** | **Median (IQR)** |  |  |
| ADA | 6.44 (6.06-6.76) | 6.38 (6.02-6.73) | 0.2526 | 0.3041 |
| AXIN1 | 3.80 (2.89-4.36) | 3.60 (2.49-4.26) | **0.0197** | **0.0331** |
| Beta-NGF | 0.61 (0.56-0.74) | 0.61 (0.43-0.77) | 0.3371 | 0.3916 |
| CASP-8 | 6.29 (5.31-7.44) | 5.93 (5.02-6.85) | **0.0012** | **0.0037** |
| CCL3 | 7.17 (6.60-7.96) | 7.03 (6.49-7.77) | 0.0508 | 0.0704 |
| CCL4 | 7.36 (6.89-8.12) | 7.25 (6.71-7.87) | **0.0363** | 0.0522 |
| CCL11 | 8.67 (8.29-9.01) | 8.54 (8.15-8.89) | **0.0007** | **0.0031** |
| CCL19 | 9.28 (8.60-9.82) | 8.84 (8.31-9.54) | **0.0002** | **0.0010** |
| CCL20 | 6.81 (6.04-7.61) | 6.44 (5.76-7.27) | **0.0018** | **0.0050** |
| CCL23 | 10.03 (9.63-10.41) | 9.86 (9.46-10.21) | **<0.0001** | **0.0004** |
| CCL25 | 6.51 (6.14-6.92) | 6.39 (5.94-6.83) | **0.0099** | **0.0190** |
| CCL28 | 2.83 (2.54-3.24) | 2.70 (2.42-3.00) | **<0.0001** | **0.0004** |
| CD5 | 5.99 (5.70-6.25) | 5.84 (5.54-6.14) | **0.0003** | **0.0013** |
| CD6 | 7.43 (7.05-7.86) | 7.34 (6.83-7.73) | **0.0027** | **0.0069** |
| CD40 | 12.55 (12.18-13.03) | 12.44 (11.98-12.89) | **0.0040** | **0.0093** |
| CD244 | 6.76 (6.45-7.10) | 6.63 (6.34-6.89) | **0.0001** | **0.0009** |
| CD8A | 9.45 (8.80-10.00) | 9.33 (8.73-9.91) | 0.1043 | 0.1350 |
| CDCP1 | 3.94 (3.40-4.40) | 3.64 (3.15-4.14) | **0.0001** | **0.0009** |
| CSF-1 | 11.02 (10.82-11.16) | 10.93 (10.73-11.12) | **0.0029** | **0.0070** |
| CST5 | 7.25 (6.95-7.65) | 7.15 (6.81-7.50) | **0.0009** | **0.0031** |
| CX3CL1 | 4.73 (4.46-5.03) | 4.57 (4.20-4.88) | **<0.0001** | **0.0001** |
| CXCL1 | 10.38 (9.88-10.96) | 10.31 (9.84-10.74) | 0.0872 | 0.1148 |
| CXCL5 | 12.63 (12.09-13.25) | 12.44 (11.75-13.03) | **0.0008** | **0.0031** |
| CXCL6 | 9.39 (8.95-9.91) | 9.24 (8.73-9.70) | **0.0045** | **0.0101** |
| CXCL9 | 7.36 (6.87-7.91) | 6.98 (6.49-7.54) | **<0.0001** | **<0.0001** |
| CXCL10 | 9.90 (9.43-10.61) | 9.72 (9.26-10.24) | **0.0012** | **0.0037** |
| CXCL11 | 8.39 (7.69-8.99) | 8.06 (7.46-8.69) | **0.0008** | **0.0031** |
| DNER | 9.67 (9.40-9.84) | 9.56 (9.32-9.77) | **0.0021** | **0.0057** |
| EN-RAGE | 7.33 (6.56-8.17) | 7.00 (6.01-7.77) | **<0.0001** | **0.0005** |
| FGF-19 | 8.82 (8.09-9.61) | 8.53 (7.82-9.24) | **0.0017** | **0.0050** |
| FGF-21 | 5.93 (5.00-6.70) | 5.65 (4.82-6.51) | **0.0478** | 0.0674 |
| FGF-23 | 0.93 (0.79-1.04) | 0.93 (0.64-1.15) | 0.6932 | 0.7301 |
| Flt3L | 9.76 (9.42-10.11) | 9.65 (9.33-9.98) | **0.0050** | **0.0107** |
| GDNF | 1.44 (1.29-1.99) | 1.43 (1.29-1.90) | **0.0253** | **0.0391** |
| HGF | 9.89 (9.49-10.26) | 9.73 (9.29-10.10) | **0.0006** | **0.0027** |
| IL-6 | 2.52 (1.90-3.60) | 2.30 (1.71-3.17) | **0.0139** | **0.0249** |
| IL-7 | 3.05 (2.48-3.44) | 3.09 (2.53-3.63) | 0.4597 | 0.5114 |
| IL-8 | 9.38 (7.64-12.41) | 9.19 (7.56-11.68) | 0.4592 | 0.5114 |
| IL-10 | 3.48 (3.08-3.84) | 3.36 (3.00-3.76) | **0.0331** | **0.0484** |
| IL-18 | 9.02 (8.64-9.45) | 8.90 (8.48-9.37) | **0.0134** | **0.0247** |
| IL-10RA | 0.73 (0.50-1.07) | 0.66 (0.43-0.98) | **0.0322** | **0.0479** |
| IL-10RB | 6.77 (6.48-6.93) | 6.64 (6.34-6.87) | **0.0001** | **0.0008** |
| IL-15RA | 1.46 (1.36-1.70) | 1.38 (1.28-1.67) | **0.0244** | **0.0385** |
| IL-18R1 | 9.14 (8.80-9.50) | 9.07 (8.68-9.40) | **0.0151** | **0.0265** |
| LAP TGF-beta-1 | 8.30 (7.91-8.61) | 8.07 (7.73-8.39) | **<0.0001** | **0.0001** |
| LIF-R | 4.81 (4.50-5.04) | 4.71 (4.48-4.92) | **0.0008** | **0.0031** |
| MCP-1 | 12.49 (12.04-13.22) | 12.45 (12.00-12.98) | 0.1333 | 0.1699 |
| MCP-2 | 9.82 (9.29-10.15) | 9.69 (9.22-10.09) | 0.0828 | 0.1109 |
| MCP-3 | 3.57 (2.43-5.93) | 3.54 (2.46-6.13) | 0.9037 | 0.9271 |
| MCP-4 | 15.15 (14.71-15.68) | 15.04 (14.51-15.49) | **0.0173** | **0.0297** |
| MMP-1 | 15.89 (15.36-16.38) | 15.91 (15.18-16.43) | 0.5209 | 0.5561 |
| MMP-10 | 9.90 (9.48-10.30) | 9.75 (9.31-10.17) | **0.0029** | **0.0070** |
| NT-3 | 1.32 (1.13-1.59) | 1.31 (1.04-1.56) | 0.4101 | 0.4695 |
| OPG | 10.44 (10.15-10.75) | 10.26 (9.94-10.56) | **<0.0001** | **<0.0001** |
| OSM | 7.98 (6.94-9.06) | 7.87 (6.97-8.91) | 0.5066 | 0.5482 |
| PD-L1 | 6.25 (5.98-6.54) | 6.10 (5.82-6.38) | **<0.0001** | **0.0001** |
| SCF | 9.72 (9.38-9.99) | 9.59 (9.24-9.86) | **0.0002** | **0.0010** |
| SIRT2 | 4.52 (3.79-5.18) | 4.38 (3.49-5.10) | **0.0301** | **0.0458** |
| SLAMF1 | 2.90 (2.51-3.31) | 2.87 (2.47-3.21) | 0.3032 | 0.3575 |
| ST1A1 | 4.37 (3.30-5.04) | 4.02 (2.87-4.81) | **0.0074** | **0.0151** |
| STAMBP | 5.82 (5.22-6.21) | 5.65 (5.05-6.11) | **0.0093** | **0.0184** |
| TGF-alpha | 4.92 (4.48-5.33) | 4.83 (4.30-5.30) | 0.0555 | 0.0756 |
| TNFB | 5.39 (5.08-5.69) | 5.35 (4.98-5.66) | 0.2404 | 0.2967 |
| TNFRSF9 | 7.60 (7.35-7.95) | 7.44 (7.11-7.78) | **<0.0001** | **0.0003** |
| TNFSF14 | 7.90 (7.27-8.49) | 7.71 (6.94-8.33) | **0.0102** | **0.0193** |
| TRAIL | 8.43 (8.13-8.63) | 8.33 (8.05-8.57) | **0.0054** | **0.0113** |
| TRANCE | 5.32 (4.92-5.79) | 5.31 (4.80-5.75) | 0.2541 | 0.3041 |
| TWEAK | 9.30 (8.94-9.58) | 9.16 (8.87-9.43) | **0.0025** | **0.0065** |
| VEGF-A | 12.36 (11.87-12.76) | 12.17 (11.69-12.63) | **0.0012** | **0.0037** |
| 4E-BP1 | 8.07 (7.29-8.68) | 7.83 (6.90-8.60) | **0.0219** | **0.0361** |
| uPA | 9.97 (9.67-10.30) | 9.84 (9.53-10.11) | **<0.0001** | **0.0004** |

Abbreviations: IQR, interquartile range; FDR, false discovery rate; For biomarker abbreviations, see Supplemental Table 1

NOTE: Numbers printed in bold are statistically significant (p < 0.05).

^a^Results of a Wilcoxon rank-sum test. Study participants with other (e.g. VD) or unknown dementia forms were excluded.

^b^P-values corrected for multiple testing by Benjamini and Hochberg method.

Supplemental Table 6. Comparison of median Olink Inflammation panel biomarker levels between vascular dementia cases (n=195) and controls (n=1278)

| **Olink Biomarker** | **Vascular dementia (n=195)** | **Controls (n=1278)** | **p-value^a^** | **FDR^b^** |
| --- | --- | --- | --- | --- |
|  | **Median (IQR)** | **Median (IQR)** |  |  |
| ADA | 6.45 (6.06-6.75) | 6.38 (6.02-6.73) | 0.2154 | 0.2701 |
| AXIN1 | 3.70 (2.39-4.37) | 3.60 (2.49-4.26) | 0.5743 | 0.6312 |
| Beta-NGF | 0.61 (0.59-0.67) | 0.61 (0.43-0.77) | 0.4956 | 0.5758 |
| CASP-8 | 6.04 (5.12-7.10) | 5.93 (5.02-6.85) | 0.2717 | 0.3302 |
| CCL3 | 7.30 (6.76-8.11) | 7.03 (6.49-7.77) | **0.0006** | **0.0015** |
| CCL4 | 7.53 (6.88-8.10) | 7.25 (6.71-7.87) | **0.0025** | **0.0050** |
| CCL11 | 8.68 (8.27-9.06) | 8.54 (8.15-8.89) | **<0.0001** | **0.0003** |
| CCL19 | 9.06 (8.44-9.73) | 8.84 (8.31-9.54) | **0.0114** | **0.0166** |
| CCL20 | 6.79 (6.03-7.74) | 6.44 (5.76-7.27) | **<0.0001** | **0.0002** |
| CCL23 | 9.98 (9.63-10.44) | 9.86 (9.46-10.21) | **<0.0001** | **0.0004** |
| CCL25 | 6.63 (6.09-7.06) | 6.39 (5.94-6.83) | **0.0001** | **0.0004** |
| CCL28 | 2.82 (2.55-3.07) | 2.70 (2.42-3.00) | **0.0005** | **0.0014** |
| CD5 | 6.06 (5.71-6.31) | 5.84 (5.54-6.14) | **<0.0001** | **<0.0001** |
| CD6 | 7.45 (6.97-7.78) | 7.34 (6.83-7.73) | 0.0529 | 0.0733 |
| CD40 | 12.62 (12.12-13.03) | 12.44 (11.98-12.89) | **0.0069** | **0.0106** |
| CD244 | 6.77 (6.44-7.09) | 6.63 (6.34-6.89) | **<0.0001** | **0.0002** |
| CD8A | 9.49 (8.95-10.11) | 9.33 (8.73-9.91) | **0.0057** | **0.0091** |
| CDCP1 | 4.01 (3.47-4.61) | 3.64 (3.15-4.14) | **<0.0001** | **<0.0001** |
| CSF-1 | 11.03 (10.85-11.19) | 10.93 (10.73-11.12) | **<0.0001** | **0.0004** |
| CST5 | 7.28 (6.94-7.64) | 7.15 (6.81-7.50) | **0.0008** | **0.0019** |
| CX3CL1 | 4.71 (4.42-5.05) | 4.57 (4.20-4.88) | **<0.0001** | **<0.0001** |
| CXCL1 | 10.48 (10.07-10.89) | 10.31 (9.84-10.74) | **0.0027** | **0.0051** |
| CXCL5 | 12.69 (12.04-13.21) | 12.44 (11.75-13.03) | **0.0015** | **0.0032** |
| CXCL6 | 9.38 (8.99-9.91) | 9.24 (8.73-9.70) | **0.0006** | **0.0015** |
| CXCL9 | 7.34 (6.89-7.98) | 6.98 (6.49-7.54) | **<0.0001** | **<0.0001** |
| CXCL10 | 10.00 (9.50-10.73) | 9.72 (9.26-10.24) | **<0.0001** | **<0.0001** |
| CXCL11 | 8.30 (7.68-8.89) | 8.06 (7.46-8.69) | **0.0015** | **0.0032** |
| DNER | 9.64 (9.39-9.87) | 9.56 (9.32-9.77) | **0.0019** | **0.0040** |
| EN-RAGE | 7.22 (6.48-8.18) | 7.00 (6.01-7.77) | **0.0002** | **0.0005** |
| FGF-19 | 8.91 (8.02-9.48) | 8.53 (7.82-9.24) | **0.002** | **0.0041** |
| FGF-21 | 5.94 (5.18-6.71) | 5.65 (4.82-6.51) | **0.0028** | **0.0053** |
| FGF-23 | 0.93 (0.79-1.02) | 0.93 (0.64-1.15) | 0.8527 | 0.8749 |
| Flt3L | 9.78 (9.47-10.15) | 9.65 (9.33-9.98) | **0.0002** | **0.0005** |
| GDNF | 1.42 (1.29-2.06) | 1.43 (1.29-1.90) | **0.0063** | **0.0100** |
| HGF | 9.97 (9.50-10.26) | 9.73 (9.29-10.10) | **<0.0001** | **<0.0001** |
| IL-6 | 2.57 (1.99-3.43) | 2.30 (1.71-3.17) | **0.0012** | **0.0026** |
| IL-7 | 3.28 (2.75-3.78) | 3.09 (2.53-3.63) | **0.0031** | **0.0056** |
| IL-8 | 9.42 (7.98-12.28) | 9.19 (7.56-11.68) | 0.1985 | 0.2529 |
| IL-10 | 3.53 (3.11-3.97) | 3.36 (3.00-3.76) | **0.0006** | **0.0015** |
| IL-18 | 9.16 (8.69-9.55) | 8.90 (8.48-9.37) | **<0.0001** | **0.0002** |
| IL-10RA | 0.71 (0.47-1.10) | 0.66 (0.43-0.98) | 0.0681 | 0.0928 |
| IL-10RB | 6.74 (6.48-7.01) | 6.64 (6.34-6.87) | **<0.0001** | **0.0004** |
| IL-15RA | 1.55 (1.36-1.73) | 1.38 (1.28-1.67) | **<0.0001** | **0.0003** |
| IL-18R1 | 9.20 (8.87-9.56) | 9.07 (8.68-9.40) | **<0.0001** | **0.0002** |
| LAP TGF-beta-1 | 8.20 (7.89-8.50) | 8.07 (7.73-8.39) | **0.0001** | **0.0004** |
| LIF-R | 4.83 (4.62-5.10) | 4.71 (4.48-4.92) | **<0.0001** | **<0.0001** |
| MCP-1 | 12.54 (11.99-13.21) | 12.45 (12.00-12.98) | 0.0722 | 0.0967 |
| MCP-2 | 9.92 (9.38-10.30) | 9.69 (9.22-10.09) | **0.0003** | **0.0009** |
| MCP-3 | 3.85 (2.65-6.56) | 3.54 (2.46-6.13) | 0.1077 | 0.1419 |
| MCP-4 | 15.15 (14.70-15.68) | 15.04 (14.51-15.49) | **0.0049** | **0.0082** |
| MMP-1 | 15.91 (15.27-16.36) | 15.91 (15.18-16.43) | 0.9038 | 0.9154 |
| MMP-10 | 9.95 (9.57-10.42) | 9.75 (9.31-10.17) | **<0.0001** | **<0.0001** |
| NT-3 | 1.40 (1.13-1.65) | 1.31 (1.04-1.56) | **0.0044** | **0.0075** |
| OPG | 10.48 (10.15-10.79) | 10.26 (9.94-10.56) | **<0.0001** | **<0.0001** |
| OSM | 7.77 (7.09-9.10) | 7.87 (6.97-8.91) | 0.7958 | 0.8382 |
| PD-L1 | 6.27 (5.95-6.58) | 6.10 (5.82-6.38) | **<0.0001** | **<0.0001** |
| SCF | 9.70 (9.32-9.99) | 9.59 (9.24-9.86) | **0.0050** | **0.0082** |
| SIRT2 | 4.51 (3.45-5.08) | 4.38 (3.49-5.10) | 0.9709 | 0.9709 |
| SLAMF1 | 2.93 (2.66-3.34) | 2.87 (2.47-3.21) | **0.0096** | **0.0143** |
| ST1A1 | 4.13 (2.70-4.85) | 4.02 (2.87-4.81) | 0.5753 | 0.6312 |
| STAMBP | 5.71 (5.04-6.13) | 5.65 (5.05-6.11) | 0.6045 | 0.6541 |
| TGF-alpha | 4.95 (4.49-5.51) | 4.83 (4.30-5.30) | **0.0192** | **0.0275** |
| TNFB | 5.35 (5.01-5.62) | 5.35 (4.98-5.66) | 0.8368 | 0.8698 |
| TNFRSF9 | 7.63 (7.29-8.01) | 7.44 (7.11-7.78) | **<0.0001** | **<0.0001** |
| TNFSF14 | 7.89 (6.87-8.49) | 7.71 (6.94-8.33) | 0.0518 | 0.0731 |
| TRAIL | 8.38 (8.14-8.68) | 8.33 (8.05-8.57) | **0.0030** | **0.0055** |
| TRANCE | 5.29 (4.83-5.86) | 5.31 (4.80-5.75) | 0.3947 | 0.4725 |
| TWEAK | 9.25 (8.92-9.54) | 9.16 (8.87-9.43) | **0.0033** | **0.0058** |
| VEGF-A | 12.45 (11.87-12.95) | 12.17 (11.69-12.63) | **<0.0001** | **<0.0001** |
| 4E-BP1 | 8.00 (7.21-8.65) | 7.83 (6.90-8.60) | 0.2272 | 0.2805 |
| uPA | 9.94 (9.66-10.28) | 9.84 (9.53-10.11) | **0.0003** | **0.0009** |

Abbreviations: IQR, interquartile range; FDR, false discovery rate; For biomarker abbreviations, see Supplemental Table 1

NOTE: Numbers printed in bold are statistically significant (p < 0.05).

^a^Results of a Wilcoxon rank-sum test. Study participants with other (e.g. AD) or unknown dementia forms were excluded.

^b^P-values corrected for multiple testing by Benjamini and Hochberg method.

Supplemental Table 7. Non-significant associations of Olink Inflammation panel biomarker levels with all-cause dementia incidence (FDR ≥ 0.05)

| **Olink Biomarker** | **Value of 1 SD** | **All-cause dementia (n=504 cases)** | | |
| --- | --- | --- | --- | --- |
|  |  | **OR (95% CI)  per 1 SD^a^** | **p-value  per 1 SD** | **FDR corrected  p-value^b^** |
| Beta-NGF | 0.397 | 0.97 (0.86-1.11) | 0.6887 | 0.6984 |
| CD8A | 0.944 | 1.13 (1.00-1.27) | **0.0492** | 0.0581 |
| FGF-21 | 1.315 | 1.11 (0.99-1.25) | 0.0716 | 0.0818 |
| FGF-23 | 0.449 | 0.99 (0.88-1.11) | 0.8587 | 0.8587 |
| IL-6 | 1.871 | 1.10 (0.98-1.23) | 0.1092 | 0.1210 |
| IL-8 | 2.570 | 1.09 (0.97-1.22) | 0.1471 | 0.1605 |
| IL-12B | 0.795 | 1.10 (0.98-1.24) | 0.1086 | 0.1210 |
| MCP-1 | 1.001 | 1.12 (1.00-1.26) | **0.0469** | 0.0563 |
| MCP-3 | 2.846 | 1.05 (0.94-1.18) | 0.3626 | 0.3784 |
| MMP-1 | 0.981 | 1.04 (0.93-1.18) | 0.4651 | 0.4784 |
| OSM | 1.501 | 1.08 (0.96-1.21) | 0.2165 | 0.2292 |
| SLAMF1 | 0.633 | 1.12 (1.00-1.27) | 0.0506 | 0.0588 |
| TNFB | 0.592 | 1.08 (0.96-1.22) | 0.1823 | 0.1959 |
| 4E-BP1 | 1.300 | 1.13 (1.00-1.27) | **0.0457** | 0.0558 |

Abbreviations: SD, standard deviation; CI, confidence interval; FDR, false discovery rate; For biomarker abbreviations, see Supplemental Table 1.

^a^ Multivariate logistic regression model adjusted for age (continuously), sex, education, physical activity, BMI (categorical), CVD, diabetes, depression, *APOE* genotype.

^b^ P-values corrected for multiple testing by the Benjamini and Hochberg method.

Supplemental Table 8. Non-significant associations of Olink Inflammation panel biomarker levels with Alzheimer’s disease incidence (FDR ≥ 0.05)

| **Olink Biomarker** | **Value of 1 SD** | **Alzheimer’s disease (n=163 cases)** | | |  |
| --- | --- | --- | --- | --- | --- |
|  |  | **OR (95% CI)  per 1 SD^a^** | **p-value  per 1 SD** | **FDR corrected  p-value^b^** | |
| ADA | 0.635 | 1.07 (0.90-1.28) | 0.4191 | 0.4572 |  |
| AXIN1 | 1.140 | 1.24 (1.04-1.50) | **0.0196** | 0.0564 |  |
| Beta-NGF | 0.397 | 0.98 (0.81-1.18) | 0.8200 | 0.8315 |  |
| CCL3 | 1.508 | 1.11 (0.94-1.31) | 0.2045 | 0.2727 |  |
| CCL4 | 1.099 | 1.16 (0.98-1.38) | 0.0792 | 0.1326 |  |
| CCL11 | 0.697 | 1.26 (1.03-1.53) | **0.0215** | 0.0594 |  |
| CCL19 | 1.199 | 1.24 (1.04-1.47) | **0.0162** | 0.0501 |  |
| CCL20 | 1.540 | 1.17 (1.00-1.38) | 0.0520 | 0.0891 |  |
| CCL25 | 0.763 | 1.08 (0.90-1.30) | 0.4293 | 0.4613 |  |
| CD5 | 0.523 | 1.24 (1.03-1.49) | **0.0253** | 0.0594 |  |
| CD40 | 0.734 | 1.24 (1.03-1.50) | **0.0259** | 0.0594 |  |
| CD8A | 0.944 | 1.09 (0.91-1.32) | 0.3392 | 0.3939 |  |
| CDCP1 | 0.894 | 1.16 (0.96-1.40) | 0.1324 | 0.1945 |  |
| CSF-1 | 0.425 | 1.22 (1.00-1.50) | 0.0512 | 0.0891 |  |
| CST5 | 0.698 | 1.25 (1.03-1.51) | **0.0226** | 0.0594 |  |
| CXCL1 | 0.901 | 1.15 (0.97-1.37) | 0.1021 | 0.1598 |  |
| CXCL9 | 0.953 | 1.23 (1.02-1.48) | **0.0264** | 0.0594 |  |
| CXCL10 | 0.953 | 1.09 (0.91-1.31) | 0.3288 | 0.3881 |  |
| CXCL11 | 1.051 | 1.21 (1.01-1.45) | **0.0341** | 0.0701 |  |
| FGF-19 | 1.089 | 1.21 (1.02-1.45) | **0.0328** | 0.0695 |  |
| FGF-21 | 1.315 | 1.10 (0.91-1.32) | 0.3247 | 0.3881 |  |
| FGF-23 | 0.449 | 0.87 (0.72-1.06) | 0.1694 | 0.2301 |  |
| Flt3L | 0.629 | 1.18 (0.98-1.41) | 0.0875 | 0.1400 |  |
| GDNF | 0.506 | 1.06 (0.88-1.28) | 0.5480 | 0.5802 |  |
| IL-6 | 1.871 | 1.10 (0.93-1.30) | 0.2483 | 0.3136 |  |
| IL-7 | 0.798 | 0.97 (0.81-1.16) | 0.7445 | 0.7658 |  |
| IL-8 | 2.570 | 1.07 (0.90-1.28) | 0.4121 | 0.4565 |  |
| IL-10 | 0.863 | 1.08 (0.91-1.29) | 0.3790 | 0.4331 |  |
| IL-18 | 0.763 | 1.22 (1.01-1.48) | **0.0393** | 0.0765 |  |
| IL-12B | 0.795 | 1.15 (0.96-1.38) | 0.1361 | 0.1960 |  |
| IL-10RA | 0.788 | 1.13 (0.96-1.33) | 0.1555 | 0.2195 |  |
| IL-15RA | 0.359 | 1.12 (0.93-1.35) | 0.2414 | 0.3104 |  |
| IL-18R1 | 0.602 | 1.18 (0.98-1.42) | 0.0862 | 0.1400 |  |
| MCP-1 | 1.001 | 1.13 (0.95-1.34) | 0.1663 | 0.2301 |  |
| MCP-2 | 0.769 | 1.08 (0.90-1.31) | 0.3892 | 0.4379 |  |
| MCP-3 | 2.846 | 0.99 (0.84-1.18) | 0.9418 | 0.9418 |  |
| MCP-4 | 0.927 | 1.22 (1.00-1.49) | **0.0465** | 0.0873 |  |
| MMP-1 | 0.981 | 1.10 (0.92-1.32) | 0.2830 | 0.3512 |  |
| MMP-10 | 0.761 | 1.21 (1.01-1.45) | **0.0393** | 0.0765 |  |
| NT-3 | 0.544 | 1.10 (0.92-1.31) | 0.2878 | 0.3512 |  |
| OPG | 0.609 | 1.28 (1.05-1.57) | **0.0167** | 0.0501 |  |
| OSM | 1.501 | 1.12 (0.94-1.33) | 0.2121 | 0.2777 |  |
| SCF | 0.624 | 1.26 (1.03-1.56) | **0.0277** | 0.0604 |  |
| SIRT2 | 1.157 | 1.20 (1.00-1.43) | 0.0517 | 0.0891 |  |
| SLAMF1 | 0.633 | 1.05 (0.87-1.27) | 0.5886 | 0.6142 |  |
| TNFB | 0.592 | 1.17 (0.96-1.41) | 0.1144 | 0.1716 |  |
| TNFRSF9 | 0.636 | 1.24 (1.03-1.50) | **0.0250** | 0.0594 |  |
| TNFSF14 | 1.064 | 1.24 (1.03-1.49) | **0.0245** | 0.0594 |  |
| TRANCE | 0.753 | 1.21 (1.00-1.45) | **0.0473** | 0.0873 |  |
| 4E-BP1 | 1.300 | 1.16 (0.97-1.39) | 0.1135 | 0.1716 |  |

Abbreviations: SD, standard deviation; CI, confidence interval; FDR, false discovery rate; For biomarker abbreviations, see Supplemental Table 1.

^a^ Multivariate logistic regression model adjusted for age (continuously), sex, education, physical activity, BMI (categorical), CVD, diabetes, depression, *APOE* genotype. Study participants with other (e.g. VD) or unknown dementia forms were excluded.

^b^ P-values corrected for multiple testing by the Benjamini and Hochberg method.

Supplemental Table 9. Non-significant associations of Olink Inflammation panel biomarker levels with vascular dementia incidence (FDR ≥ 0.05)

| **Olink Biomarker** | **Value of 1 SD** | **Vascular dementia (n=195 cases)** | | |
| --- | --- | --- | --- | --- |
|  |  | **OR (95% CI)  per 1 SD^a^** | **p-value  per 1 SD** | **FDR corrected  p-value^b^** |
| ADA | 0.635 | 1.08 (0.92-1.27) | 0.3647 | 0.4376 |
| AXIN1 | 1.140 | 1.06 (0.90-1.25) | 0.4768 | 0.5364 |
| Beta-NGF | 0.397 | 0.98 (0.83-1.16) | 0.8471 | 0.8839 |
| CASP-8 | 1.364 | 1.08 (0.92-1.28) | 0.3416 | 0.4198 |
| CCL3 | 1.508 | 1.17 (1.00-1.36) | **0.0473** | 0.0811 |
| CCL4 | 1.099 | 1.19 (1.01-1.39) | **0.0329** | 0.0623 |
| CCL19 | 1.199 | 1.08 (0.92-1.27) | 0.3440 | 0.4198 |
| CCL20 | 1.540 | 1.19 (1.02-1.39) | **0.0260** | 0.0520 |
| CCL25 | 0.763 | 1.18 (1.00-1.41) | 0.0564 | 0.0864 |
| CCL28 | 0.548 | 1.17 (1.00-1.38) | 0.0503 | 0.0823 |
| CD6 | 0.757 | 1.15 (0.97-1.37) | 0.0966 | 0.1419 |
| CD40 | 0.734 | 1.18 (1.00-1.40) | 0.0555 | 0.0864 |
| CD8A | 0.944 | 1.14 (0.96-1.35) | 0.1316 | 0.1858 |
| CSF-1 | 0.425 | 1.24 (1.03-1.49) | **0.0240** | 0.0508 |
| CST5 | 0.698 | 1.08 (0.91-1.27) | 0.3916 | 0.4622 |
| CXCL11 | 1.051 | 1.21 (1.02-1.43) | **0.0250** | 0.0514 |
| FGF-19 | 1.089 | 1.18 (1.01-1.39) | **0.0404** | 0.0727 |
| FGF-21 | 1.315 | 1.14 (0.96-1.35) | 0.1277 | 0.1839 |
| FGF-23 | 0.449 | 0.96 (0.83-1.12) | 0.6277 | 0.6745 |
| GDNF | 0.506 | 1.16 (0.99-1.35) | 0.0655 | 0.0983 |
| IL-6 | 1.871 | 1.11 (0.95-1.30) | 0.1709 | 0.2322 |
| IL-8 | 2.570 | 1.11 (0.94-1.30) | 0.2125 | 0.2732 |
| IL-12B | 0.795 | 1.13 (0.96-1.34) | 0.1397 | 0.1934 |
| IL-10RA | 0.788 | 1.16 (1.00-1.34) | 0.0501 | 0.0823 |
| IL-10RB | 0.533 | 1.21 (1.01-1.44) | **0.0372** | 0.0687 |
| IL-15RA | 0.359 | 1.20 (1.02-1.42) | **0.0278** | 0.0541 |
| MCP-1 | 1.001 | 1.11 (0.95-1.30) | 0.2037 | 0.2667 |
| MCP-3 | 2.846 | 1.06 (0.90-1.24) | 0.5008 | 0.5547 |
| MMP-1 | 0.981 | 1.00 (0.86-1.17) | 0.9633 | 0.9769 |
| OSM | 1.501 | 1.07 (0.91-1.26) | 0.4036 | 0.4687 |
| SCF | 0.624 | 1.12 (0.94-1.32) | 0.1974 | 0.2632 |
| SIRT2 | 1.157 | 1.00 (0.85-1.18) | 0.9941 | 0.9941 |
| SLAMF1 | 0.633 | 1.07 (0.91-1.26) | 0.4175 | 0.4771 |
| ST1A1 | 1.304 | 1.09 (0.92-1.29) | 0.3037 | 0.3836 |
| STAMBP | 0.833 | 1.04 (0.88-1.22) | 0.6686 | 0.7079 |
| TNFB | 0.592 | 1.05 (0.89-1.24) | 0.5723 | 0.6243 |
| TNFSF14 | 1.064 | 1.18 (1.00-1.39) | 0.0546 | 0.0864 |
| TRANCE | 0.753 | 1.19 (1.00-1.40) | **0.0455** | 0.0799 |
| 4E-BP1 | 1.300 | 1.00 (0.85-1.19) | 0.9593 | 0.9769 |

Abbreviations: SD, standard deviation; CI, confidence interval; FDR, false discovery rate; For biomarker abbreviations, see Supplemental Table 1.

^a^ Multivariate logistic regression model adjusted for age (continuously), sex, education, physical activity, BMI (categorical), CVD, diabetes, depression, *APOE* genotype. Study participants with other (e.g. AD) or unknown dementia forms were excluded.

^b^ P-values corrected for multiple testing by the Benjamini and Hochberg method.

## Supplemental Table 10. Replication of strong associations of Olink Inflammation panel biomarker levels with all-cause dementia incidence with multivariate, weighted Cox proportional hazards regression.

| **Olink Biomarker** | **Value of 1 SD** | **All-cause dementia (n=504 cases)** | | |
| --- | --- | --- | --- | --- |
|  |  | **HR (95% CI)  per 1 SD^*^** | **p-value  per 1 SD** | **FDR corrected  p-value**^†^ |
| CD244 | 0.587 | 1.41 (1.16-1.70) | **0.0004** | **0.0028** |
| CX3CL1 | 0.669 | 1.59 (1.30-1.95) | **<0.0001** | **0.0002** |
| CXCL5 | 0.957 | 1.26 (1.07-1.48) | **0.0050** | **0.0157** |
| DNER | 0.488 | 1.35 (1.08-1.67) | **0.0070** | **0.0201** |
| EN-RAGE | 1.307 | 1.41 (1.21-1.65) | **<0.0001** | **0.0002** |
| HGF | 0.719 | 1.50 (1.24-1.80) | **<0.0001** | **0.0002** |
| IL-18 | 0.763 | 1.34 (1.14-1.59) | **0.0005** | **0.0030** |
| LAP TGF-beta-1 | 0.574 | 1.41 (1.21-1.65) | **<0.0001** | **0.0002** |
| LIF-R | 0.503 | 1.45 (1.18-1.79) | **0.0004** | **0.0026** |
| OPG | 0.609 | 1.58 (1.28-1.96) | **<0.0001** | **0.0002** |
| TRAIL | 0.513 | 1.26 (1.05-1.52) | **0.0150** | **0.0360** |
| TWEAK | 0.647 | 1.46 (1.15-1.85) | **0.0022** | **0.0097** |
| VEGF-A | 0.794 | 1.62 (1.35-1.93) | **<0.0001** | **<0.0001** |
| uPA | 0.604 | 1.31 (1.10-1.57) | **0.0028** | **0.0109** |

Abbreviations: SD, standard deviation; HR, hazard ratio; CI, confidence interval; FDR, false discovery rate; For biomarker abbreviations, see Supplemental Table 1.

Notes: Numbers printed in bold are statistically significant (p < 0.05).

^*^ Weighted Cox regression model adjusted for age (continuously), sex, education, physical activity, BMI (categorical), CVD, diabetes, depression, *APOE* genotype.

^†^ P-values corrected for multiple testing by the Benjamini and Hochberg method.

## Supplemental Table 11. Replication of strong associations of Olink Inflammation panel biomarker levels with Alzheimer’s disease incidence with multivariate, weighted Cox proportional hazards regression.

| **Olink Biomarker** | **Value of 1 SD** | **Alzheimer’s disease (n=163 cases)** | | |
| --- | --- | --- | --- | --- |
|  |  | **HR (95% CI)  per 1 SD^*^** | **p-value  per 1 SD** | **FDR corrected  p-value**^†^ |
| CASP-8 | 1.364 | 1.20 (0.94-1.54) | 0.1468 | 0.2935 |
| CCL23 | 0.732 | 1.51 (1.10-2.07) | **0.0107** | 0.0592 |
| CCL28 | 0.548 | 1.38 (1.10-1.75) | **0.0062** | 0.0555 |
| CD244 | 0.587 | 1.49 (1.08-2.07) | **0.0162** | 0.0735 |
| CX3CL1 | 0.669 | 1.47 (1.09-1.97) | **0.0107** | 0.0592 |
| CXCL5 | 0.957 | 1.40 (1.08-1.83) | **0.0126** | 0.0651 |
| CXCL6 | 0.848 | 1.12 (0.87-1.44) | 0.3974 | 0.4933 |
| DNER | 0.488 | 1.35 (0.94-1.95) | 0.1067 | 0.2542 |
| EN-RAGE | 1.307 | 1.62 (1.23-2.12) | **0.0005** | **0.0180** |
| HGF | 0.719 | 1.67 (1.21-2.30) | **0.0018** | **0.0245** |
| IL-10RB | 0.533 | 1.32 (0.95-1.85) | 0.1017 | 0.2542 |
| LAP TGF-beta-1 | 0.574 | 1.61 (1.27-2.04) | **<0.0001** | **0.0051** |
| LIF-R | 0.503 | 1.39 (1.00-1.95) | 0.0522 | 0.1504 |
| PD-L1 | 0.6 | 1.39 (1.13-1.72) | **0.0020** | **0.0245** |
| TWEAK | 0.647 | 1.59 (1.01-2.51) | **0.0459** | 0.1400 |
| VEGF-A | 0.794 | 1.61 (1.20-2.14) | **0.0012** | **0.0245** |
| uPA | 0.604 | 1.44 (1.09-1.90) | **0.0093** | 0.0592 |

Abbreviations: SD, standard deviation; HR, hazard ratio; CI, confidence interval; FDR, false discovery rate; For biomarker abbreviations, see Supplemental Table 1.

Notes: Numbers printed in bold are statistically significant (p < 0.05).

^*^ Weighted Cox regression model adjusted for age (continuously), sex, education, physical activity, BMI (categorical), CVD, diabetes, depression, *APOE* genotype.

^†^ P-values corrected for multiple testing by the Benjamini and Hochberg method.

## Supplemental Table 12. Replication of strong associations of Olink Inflammation panel biomarker levels with vascular dementia incidence with multivariate, weighted Cox proportional hazards regression.

| **Olink Biomarker** | **Value of 1 SD** | **Vascular dementia (n=195 cases)** | | |
| --- | --- | --- | --- | --- |
|  |  | **HR (95% CI)  per 1 SD^*^** | **p-value  per 1 SD** | **FDR corrected  p-value**^†^ |
| CD5 | 0.523 | 1.26 (1.04-1.52) | **0.0190** | 0.0546 |
| CD244 | 0.587 | 1.45 (1.13-1.85) | **0.0033** | **0.0171** |
| CX3CL1 | 0.669 | 1.55 (1.18-2.05) | **0.0019** | **0.0139** |
| CXCL5 | 0.957 | 1.32 (1.07-1.64) | **0.0112** | **0.0383** |
| CXCL6 | 0.848 | 1.19 (0.97-1.47) | 0.0913 | 0.1644 |
| DNER | 0.488 | 1.43 (1.04-1.96) | **0.0287** | 0.0737 |
| EN-RAGE | 1.307 | 1.46 (1.18-1.81) | **0.0004** | **0.0080** |
| HGF | 0.719 | 1.49 (1.17-1.90) | **0.0013** | **0.0131** |
| IL-18 | 0.763 | 1.40 (1.13-1.74) | **0.0019** | **0.0139** |
| LAP TGF-beta-1 | 0.574 | 1.43 (1.17-1.74) | **0.0004** | **0.0080** |
| LIF-R | 0.503 | 1.53 (1.17-2.01) | **0.0022** | **0.0145** |
| OPG | 0.609 | 1.61 (1.22-2.12) | **0.0007** | **0.0100** |
| TRAIL | 0.513 | 1.28 (1.00-1.63) | **0.0476** | 0.0927 |
| TWEAK | 0.647 | 1.56 (1.09-2.22) | **0.0143** | **0.0467** |
| VEGF-A | 0.794 | 1.68 (1.31-2.16) | **<0.0001** | **0.0021** |

Abbreviations: SD, standard deviation; HR, hazard ratio; CI, confidence interval; FDR, false discovery rate; For biomarker abbreviations, see Supplemental Table 1.

Notes: Numbers printed in bold are statistically significant (p < 0.05).

^*^ Weighted Cox regression model adjusted for age (continuously), sex, education, physical activity, BMI (categorical), CVD, diabetes, depression, *APOE* genotype.

^†^ P-values corrected for multiple testing by the Benjamini and Hochberg method.

Supplemental Table 13. Olink Inflammation panel biomarkers in the CX3CL1 cluster

| **No.** | **Biomarker** | **Spearman’s r with CX3CL1^a^** |
| --- | --- | --- |
| 1 | IL-10RB^b,c^ | 0.672 |
| 2 | LIF-R^b,c,d^ | 0.661 |
| 3 | OPG^b,d^ | 0.621 |
| 4 | CCL23^b,c,d^ | 0.603 |
| 5 | PD-L1^b,c,d^ | 0.602 |
| 6 | TNFRSF9^b,d^ | 0.600 |
| 7 | CSF-1^b^ | 0.589 |
| 8 | IL-15RA^b^ | 0.568 |
| 9 | TWEAK^b,c,d^ | 0.561 |
| 10 | CCL25^b^ | 0.555 |
| 11 | TRAIL^b,c,d^ | 0.550 |
| 12 | Flt3L^b^ | 0.546 |
| 13 | DNER^b,c,d^ | 0.537 |
| 14 | uPA^b,c,d^ | 0.523 |
| 15 | CD244^b,c,d^ | 0.522 |
| 16 | CD5^b,d^ | 0.522 |
| 17 | SCF^b^ | 0.508 |
| 18 | IL-12B | 0.506 |

^a^All biomarkers with r > 0.5 with CX3CL1 were selected for the cluster.

^b^Significantly associated with all-cause dementia.

^c^Significantly associated with Alzheimer’s disease.

^d^Significantly associated with vascular dementia.

Note: For biomarker abbreviations, see Supplemental Table 1.

## **Supplemental Table 14**. Olink Inflammation panel biomarkers in the EN-RAGE cluster

| **No.** | **Biomarker** | **Spearman’s r with EN-RAGE^a^** | |
| --- | --- | --- | --- |
| 1 | TNFSF14^b^ | 0.817 |  |
| 2 | OSM | 0.799 |  |
| 3 | CASP-8^b,c^ | 0.784 |  |
| 4 | CD40^b^ | 0.753 |  |
| 5 | STAMBP^b,c^ | 0.750 |  |
| 6 | HGF^b,c,d^ | 0.735 |  |
| 7 | SIRT2^b^ | 0.731 |  |
| 8 | TGF-alpha^b,c,d^ | 0.715 |  |
| 9 | IL-8 | 0.705 |  |
| 10 | AXIN1^b^ | 0.675 |  |
| 11 | CCL3^b^ | 0.664 |  |
| 12 | uPA^b,c,d^ | 0.643 |  |
| 13 | ADA^b^ | 0.632 |  |
| 14 | CCL4^b^ | 0.620 |  |
| 15 | CD244^b,c,d^ | 0.599 |  |
| 16 | VEGF-A^b,c,d^ | 0.593 |  |
| 17 | MCP-3 | 0.591 |  |
| 18 | CSF-1^b^ | 0.574 |  |
| 19 | ST1A1^b,c^ | 0.561 |  |
| 20 | CXCL11^b^ | 0.559 |  |
| 21 | MCP-1 | 0.552 |  |
| 22 | IL-6 | 0.542 |  |
| 23 | CD6^b,c^ | 0.536 |  |
| 24 | CD5^b,d^ | 0.535 |  |
| 25 | IL-18^b,d^ | 0.517 |  |
| 26 | CXCL1^b,d^ | 0.511 |  |

^a^All biomarkers with r > 0.5 with EN-RAGE were selected for the cluster.

^b^Significantly associated with all-cause dementia.

^c^Significantly associated with Alzheimer’s disease.

^d^Significantly associated with vascular dementia.

Note: For biomarker abbreviations, see Supplemental Table 1.

## **Supplemental Table 15**. Olink Inflammation panel biomarkers in the LAP TGF-beta-1 cluster

| **No.** | **Biomarker** | **Spearman’s r with LAP TGF-beta-1^a^** |
| --- | --- | --- |
| 1 | HGF^b,c,d^ | 0.601 |
| 2 | CD244^b,c,d^ | 0.577 |
| 3 | TWEAK^b,c,d^ | 0.560 |
| 4 | uPA^b,c,d^ | 0.557 |
| 5 | OPG^b,d^ | 0.543 |
| 6 | LIF-R^b,c,d^ | 0.540 |
| 7 | CSF-1 | 0.539 |
| 8 | IL-10RB^b,c^ | 0.539 |
| 9 | PD-L1^b,c,d^ | 0.518 |
| 10 | VEGF-A^b,d^ | 0.516 |
| 11 | DNER^b,c,d^ | 0.513 |
| 12 | CD5^b,d^ | 0.510 |
| 13 | CCL11^b,d^ | 0.509 |
| 14 | MCP-4^b,d^ | 0.504 |
| 15 | CXCL6^b,c,d^ | 0.501 |
| 16 | CD40^b^ | 0.500 |

^a^All biomarkers with r > 0.5 with LAP TGF-beta-1 were selected for the cluster.

^b^Significantly associated with all-cause dementia.

^c^Significantly associated with Alzheimer’s disease.

^d^Significantly associated with vascular dementia.

Note: For biomarker abbreviations, see Supplemental Table 1.

## **Supplemental Table 16**. Olink Inflammation panel biomarkers in the VEGF-A cluster

| **No.** | **Biomarker** | **Spearman’s r with VEGF-A^a^** |
| --- | --- | --- |
| 1 | HGF^b,c,d^ | 0.680 |
| 2 | CD40^b^ | 0.646 |
| 3 | CSF-1 | 0.620 |
| 4 | uPA^b,c,d^ | 0.608 |
| 5 | EN-RAGE^b,c,d^ | 0.593 |
| 6 | CD244^b,c,d^ | 0.584 |
| 7 | MCP-1 | 0.563 |
| 8 | OSM | 0.561 |
| 9 | CXCL1^b,d^ | 0.558 |
| 10 | CCL3^b^ | 0.556 |
| 11 | TGF-alpha^b,c,d^ | 0.554 |
| 12 | TNFSF14^b^ | 0.551 |
| 13 | IL-6 | 0.538 |
| 14 | CCL4^b^ | 0.534 |
| 15 | STAMBP^b,c^ | 0.531 |
| 16 | PD-L1^b,c,d^ | 0.529 |
| 17 | IL-10RB^b,c^ | 0.526 |
| 18 | CD5^b,d^ | 0.522 |
| 19 | TNFRSF9^b,d^ | 0.520 |
| 20 | CXCL11^b^ | 0.519 |
| 21 | IL-10^b,d^ | 0.518 |
| 22 | OPG^b,d^ | 0.518 |
| 23 | SIRT2^b^ | 0.518 |
| 24 | LAP TGF-beta-1^b,c,d^ | 0.516 |
| 25 | ADA^b^ | 0.514 |
| 26 | TWEAK^b,c,d^ | 0.504 |
| 27 | IL-18R1^b,d^ | 0.504 |
| 28 | CCL11^b,d^ | 0.502 |

^a^All biomarkers with r > 0.5 with VEGF-A were selected for the cluster.

^b^Significantly associated with all-cause dementia.

^c^Significantly associated with Alzheimer’s disease.

^d^Significantly associated with vascular dementia.

For biomarker abbreviations, see Supplemental Table 1.

## **Supplemental Table 17**. Exploratory subgroup and sensitivity analyses for the association of CX3CL1 and all-cause dementia

| **Group** | **n_total_** | **n_cases_** | **OR (95% CI)^a^** | **p-value interaction** |
| --- | --- | --- | --- | --- |
| **Total cohort** | 1782 | 504 | 1.41 (1.24-1.60) |  |
| **Stratified by age** | | | | 0.2767 |
| <68 years | 1258 | 254 | 1.51 (1.29-1.78) |  |
| ≥68 years | 524 | 250 | 1.49 (1.22-1.83) |  |
| **Stratified by sex** | | | | 0.6705 |
| Women | 965 | 262 | 1.36 (1.14-1.61) |  |
| Men | 817 | 242 | 1.48 (1.23-1.78) |  |
| **Stratified by obesity** | | | | 0.9649 |
| BMI < 30 kg/m² | 479 | 144 | 1.46 (1.14-1.87) |  |
| BMI ≥ 30 kg/m² | 1303 | 360 | 1.40 (1.21-1.62) |  |
| **Stratified by diabetes** | | | | 0.0804 |
| No | 1489 | 394 | 1.47 (1.28-1.69) |  |
| Yes | 293 | 110 | 1.19 (0.87-1.64) |  |
| **Stratified by CVD** | | | | 0.8759 |
| No | 1373 | 350 | 1.40 (1.21-1.63) |  |
| Yes | 409 | 154 | 1.45 (1.13-1.86) |  |
| **Stratified by APOE ε4** | | | | 0.5879 |
| Negative | 1276 | 313 | 1.42 (1.22-1.66) |  |
| Positive | 506 | 193 | 1.34 (1.07-1.69) |  |
| **Stratified by time of diagnosis** | | | | NA |
| In first 10 years of FUP | 1498 | 220 | 1.65 (1.36-2.00) |  |
| In year 11-19 of FUP | 1562 | 284 | 1.28 (1.10-1.48) |  |
| **Excluding subjects free of dementia who died prior to 80^th^ birthday** | | | | NA |
| No | 1782 | 504 | 1.41 (1.24-1.60) |  |
| Yes | 1601 | 504 | 1.47 (1.29-1.67) |  |
| **Excluding subjects with sign of acute infection (CRP level >20mg/L)** | | | | NA |
| No | 1782 | 504 | 1.41 (1.24-1.60) |  |
| Yes | 1745 | 497 | 1.41 (1.24-1.60) |  |

Abbreviations: CX3CL1, Fractalkine; OR, odds ratio; CI, confidence interval; CVD, cardiovascular disease; APOE, apolipoprotein E; CRP, C-reactive protein;

NOTE: Numbers printed in bold are statistically significant (p < 0.05).

^a^Results of multivariate logistic regression model adjusted for age (continuously), sex, education, physical activity, BMI (categorical), CVD, diabetes, depression, *APOE* genotype.

## **Supplemental Table 18**. Exploratory subgroup and sensitivity analyses for the association of EN-RAGE and all-cause dementia

| **Group** | **n_total_** | **n_cases_** | **OR (95% CI)^a^** | **p-value interaction** |
| --- | --- | --- | --- | --- |
| **Total cohort** | 1782 | 504 | 1.41 (1.25-1.60) |  |
| **Stratified by age** | | | | 0.3201 |
| <68 years | 1258 | 254 | 1.41 (1.21-1.64) |  |
| ≥68 years | 524 | 250 | 1.34 (1.11-1.61) |  |
| **Stratified by sex** | | | | 0.2769 |
| Women | 965 | 262 | 1.55 (1.30-1.85) |  |
| Men | 817 | 242 | 1.32 (1.11-1.56) |  |
| **Stratified by obesity** | | | | 0.2911 |
| BMI < 30 kg/m² | 479 | 144 | 1.31 (1.04-1.66) |  |
| BMI ≥ 30 kg/m² | 1303 | 360 | 1.48 (1.28-1.71) |  |
| **Stratified by diabetes** | | | | 0.3445 |
| No | 1489 | 394 | 1.45 (1.27-1.66) |  |
| Yes | 293 | 110 | 1.31 (0.99-1.74) |  |
| **Stratified by CVD** | | | | 0.9128 |
| No | 1373 | 350 | 1.43 (1.24-1.66) |  |
| Yes | 409 | 154 | 1.37 (1.09-1.73) |  |
| **Stratified by APOE ε4** | | | | **0.0241** |
| Negative | 1276 | 313 | 1.56 (1.34-1.81) |  |
| Positive | 506 | 193 | 1.16 (0.94-1.42) |  |
| **Stratified by time of diagnosis** | | | | NA |
| In first 10 years of FUP | 1498 | 220 | 1.46 (1.22-1.73) |  |
| In year 11-19 of FUP | 1562 | 284 | 1.36 (1.18-1.57) |  |
| **Excluding subjects free of dementia who died prior to 80^th^ birthday** | | | | NA |
| No | 1782 | 504 | 1.41 (1.25-1.60) |  |
| Yes | 1601 | 504 | 1.49 (1.31-1.68) |  |
| **Excluding subjects with sign of acute infection (CRP level >20mg/L)** | | | | NA |
| No | 1782 | 504 | 1.41 (1.25-1.60) |  |
| Yes | 1745 | 497 | 1.43 (1.27-1.62) |  |

Abbreviations: EN-RAGE, Protein S100-A12; OR, odds ratio; CI, confidence interval; CVD, cardiovascular disease; APOE, apolipoprotein E; CRP, C-reactive protein;

NOTE: Numbers printed in bold are statistically significant (p < 0.05).

^a^ Results of multivariate logistic regression model adjusted for age (continuously), sex, education, physical activity, BMI (categorical), CVD, diabetes, depression, *APOE* genotype.

## **Supplemental Table 19**. Exploratory subgroup and sensitivity analyses for the association of EN-RAGE and Alzheimer’s disease

| **Group** | **n_total_** | **n_cases_** | **OR (95% CI)^a^** | **p-value interaction** |
| --- | --- | --- | --- | --- |
| **Total cohort** | 1782 | 163 | 1.51 (1.25-1.83) |  |
| **Stratified by age** | | | | 0.7589 |
| <68 years | 1258 | 88 | 1.43 (1.12-1.81) |  |
| ≥68 years | 524 | 75 | 1.54 (1.12-2.11) |  |
| **Stratified by sex** | | | | 0.6325 |
| Women | 965 | 95 | 1.46 (1.14-1.88) |  |
| Men | 817 | 68 | 1.58 (1.16-2.15) |  |
| **Stratified by obesity** | | | | 0.9321 |
| BMI < 30 kg/m² | 479 | 53 | 1.53 (1.06-2.22) |  |
| BMI ≥ 30 kg/m² | 1303 | 110 | 1.50 (1.19-1.89) |  |
| **Stratified by diabetes** | | | | 0.2142 |
| No | 1489 | 131 | 1.40 (1.14-1.72) |  |
| Yes | 293 | 32 | 2.01 (1.22-3.30)^b^ |  |
| **Stratified by CVD** | | | | 0.4966 |
| No | 1373 | 124 | 1.56 (1.25-1.95) |  |
| Yes | 409 | 39 | 1.31 (0.90-1.91)^b^ |  |
| **Stratified by APOE ε4** | | | | 0.0798 |
| Negative | 1276 | 87 | 1.78 (1.35-2.33) |  |
| Positive | 506 | 76 | 1.22 (0.93-1.61) |  |
| **Stratified by time of diagnosis** | | | | NA |
| In first 10 years of FUP | 1699 | 80 | 1.79 (1.34-2.36) |  |
| In year 11-19 of FUP | 1702 | 83 | 1.31 (1.02-1.67) |  |
| **Excluding subjects free of dementia who died prior to 80^th^ birthday** | | | | NA |
| No | 1782 | 163 | 1.51 (1.25-1.83) |  |
| Yes | 1601 | 163 | 1.58 (1.30-1.92) |  |
| **Excluding subjects with sign of acute infection (CRP level >20mg/L)** | | | | NA |
| No | 1782 | 163 | 1.51 (1.25-1.83) |  |
| Yes | 1745 | 160 | 1.51 (1.24-1.83) |  |

Abbreviations: EN-RAGE, Protein S100-A12; OR, odds ratio; CI, confidence interval; CVD, cardiovascular disease; APOE, apolipoprotein E; CRP, C-reactive protein;

NOTE: Numbers printed in bold are statistically significant (p < 0.05).

^a^Results of multivariate logistic regression model adjusted for age (continuously), sex, education, physical activity, BMI (categorical), CVD, diabetes, depression, *APOE* genotype. Study participants with other (e.g. VD) or unknown dementia forms were excluded.

^b^Results of multivariate logistic regression model adjusted for age (continuously), sex, education, physical activity, BMI (categorical), CVD, diabetes. Study participants with other (e.g. VD) or unknown dementia forms were excluded.

## **Supplemental Table 20**. Exploratory subgroup and sensitivity analyses for the association of LAP TGF-beta-1 and Alzheimer’s disease

| **Group** | **n_total_** | **n_cases_** | **OR (95% CI)^a^** | **p-value interaction** |
| --- | --- | --- | --- | --- |
| **Total cohort** | 1782 | 163 | 1.46 (1.21-1.76) |  |
| **Stratified by age** | | | | 0.6941 |
| <68 years | 1258 | 88 | 1.41 (1.12-1.78) |  |
| ≥68 years | 524 | 75 | 1.49 (1.09-2.03) |  |
| **Stratified by sex** | | | | 0.9685 |
| Women | 965 | 95 | 1.51 (1.17-1.94) |  |
| Men | 817 | 68 | 1.47 (1.09-1.97) |  |
| **Stratified by obesity** | | | | 0.3398 |
| BMI < 30 kg/m² | 479 | 53 | 1.30 (0.88-1.93) |  |
| BMI ≥ 30 kg/m² | 1303 | 110 | 1.53 (1.23-1.91) |  |
| **Stratified by diabetes** | | | | 0.7049 |
| No | 1489 | 131 | 1.48 (1.20-1.84) |  |
| Yes | 293 | 32 | 1.64 (1.05-2.54)^b^ |  |
| **Stratified by CVD** | | | | 0.3792 |
| No | 1373 | 124 | 1.55 (1.25-1.93) |  |
| Yes | 409 | 39 | 1.28 (0.88-1.85)^b^ |  |
| **Stratified by APOE ε4** | | | | 0.3987 |
| Negative | 1276 | 87 | 1.59 (1.21-2.07) |  |
| Positive | 506 | 76 | 1.33 (1.02-1.74) |  |
| **Stratified by time of diagnosis** | | | | NA |
| In first 10 years of FUP | 1699 | 80 | 1.47 (1.13-1.91) |  |
| In year 11-19 of FUP | 1702 | 83 | 1.46 (1.15-1.85) |  |
| **Excluding subjects free of dementia who died prior to 80^th^ birthday** | | | | NA |
| No | 1782 | 163 | 1.46 (1.21-1.76) |  |
| Yes | 1601 | 163 | 1.56 (1.28-1.90) |  |
| **Excluding subjects with sign of acute infection (CRP level >20mg/L)** | | | | NA |
| No | 1782 | 163 | 1.46 (1.21-1.76) |  |
| Yes | 1745 | 160 | 1.46 (1.21-1.77) |  |

Abbreviations: LAP TGF-beta-1, Latency-associated peptide transforming growth factor beta-1; OR, odds ratio; CI, confidence interval; CVD, cardiovascular disease; APOE, apolipoprotein E; CRP, C-reactive protein;

NOTE: Numbers printed in bold are statistically significant (p < 0.05).

^a^Results of multivariate logistic regression model adjusted for age (continuously), sex, education, physical activity, BMI (categorical), CVD, diabetes, depression, *APOE* genotype. Study participants with other (e.g. VD) or unknown dementia forms were excluded.

^b^Results of multivariate logistic regression model adjusted for age (continuously), sex, education, physical activity, BMI (categorical), CVD, diabetes. Study participants with other (e.g. VD) or unknown dementia forms were excluded.

Supplemental Table 21. Exploratory subgroup and sensitivity analyses for the association of VEGF-A and vascular dementia

| **Group** | **n_total_** | **n_cases_** | **OR (95% CI)^a^** | **p-value interaction** |
| --- | --- | --- | --- | --- |
| **Total cohort** | 1782 | 195 | 1.43 (1.20-1.70) |  |
| **Stratified by age** | | | | 0.6534 |
| <68 years | 524 | 98 | 1.24 (1.00-1.54) |  |
| ≥68 years | 1258 | 97 | 1.75 (1.31-2.34) |  |
| **Stratified by sex** | | | | 0.7408 |
| Women | 965 | 100 | 1.41 (1.10-1.79) |  |
| Men | 817 | 95 | 1.45 (1.13-1.86) |  |
| **Stratified by obesity** | | | | 0.5126 |
| BMI < 30 kg/m² | 479 | 49 | 1.34 (0.95-1.90) |  |
| BMI ≥ 30 kg/m² | 1303 | 146 | 1.48 (1.21-1.81) |  |
| **Stratified by diabetes** | | | | 0.7305 |
| No | 1489 | 147 | 1.44 (1.19-1.74) |  |
| Yes | 293 | 48 | 1.40 (0.91-2.16) |  |
| **Stratified by CVD** | | | | 0.1381 |
| No | 1373 | 124 | 1.34 (1.08-1.65) |  |
| Yes | 409 | 71 | 1.74 (1.24-2.44) |  |
| **Stratified by APOE ε4** | | | | 0.5134 |
| Negative | 1276 | 127 | 1.47 (1.19-1.82) |  |
| Positive | 506 | 68 | 1.32 (0.97-1.81) |  |
| **Stratified by time of diagnosis** | | | | NA |
| In first 10 years of FUP | 1666 | 79 | 1.75 (1.33-2.32) |  |
| In year 11-19 of FUP | 1703 | 116 | 1.29 (1.04-1.59) |  |
| **Excluding subjects free of dementia who died prior to 80^th^ birthday** | | | | NA |
| No | 1782 | 195 | 1.43 (1.20-1.70) |  |
| Yes | 1601 | 195 | 1.52 (1.27-1.82) |  |
| **Excluding subjects with sign of acute infection (CRP level >20mg/L)** | | | | NA |
| No | 1782 | 195 | 1.43 (1.20-1.70) |  |
| Yes | 1745 | 192 | 1.46 (1.22-1.74) |  |

Abbreviations: VEGF-A, Vascular endothelial growth factor-A; OR, odds ratio; CI, confidence interval; CVD, cardiovascular disease; APOE, apolipoprotein E; CRP, C-reactive protein;

NOTE: Numbers printed in bold are statistically significant (p < 0.05).

^a^Results of multivariate logistic regression model adjusted for age (continuously), sex, education, physical activity, BMI (categorical), CVD, diabetes, depression, *APOE* genotype. Study participants with other (e.g. AD) or unknown dementia forms were excluded.

## Supplemental Table 22. Associations of five Olink inflammation panel biomarker levels with all-cause dementia, Alzheimer’s disease, and vascular dementia incidence modelled linearly and modelled with their best fitting function.

| **Linearly modelled** | | | | | |  | **Modelled with their best fitting function** | | | | |
| --- | --- | --- | --- | --- | --- | --- | --- | --- | --- | --- | --- |
| **Olink Biomarker** | **Value of 1 SD** | **OR (95% CI)  per 1 SD^*^** | | **p-value  per 1 SD** | **FDR corrected  p-value**^†^ |  | **Olink Biomarker** | **Value of 1 SD** | **OR (95% CI)  per 1 SD^*^** | **p-value  per 1 SD** | **FDR corrected  p-value**^†^ |
| **All-cause dementia (n=504 cases)** | | | | | |  |  |  |  |  |  |
| Beta-NGF | 0.397 | 0.97 (0.86-1.11) | | 0.6887 | 0.6984 |  | Beta-NGF^a^ | 39.670 | 0.82 (0.71-0.95) | **0.0065** | **0.0102** |
| CCL3 | 1.508 | 1.14 (1.02-1.27) | | **0.0259** | **0.0352** |  | CCL3^b^ | 0.008 | 0.82 (0.71-0.93) | **0.0031** | **0.0060** |
| CCL20 | 1.540 | 1.18 (1.05-1.32) | | **0.0041** | **0.0082** |  | CCL20^b^ | 0.011 | 0.79 (0.69-0.90) | **0.0005** | **0.0014** |
| GDNF | 0.506 | 1.16 (1.03-1.30) | | **0.0149** | **0.0219** |  | GDNF^b^ | 0.756 | 0.73 (0.59-0.92) | **0.0070** | **0.0105** |
| IL-10RA | 0.788 | 1.12 (1.01-1.25) | | **0.0362** | **0.0461** |  | IL-10RA^b^ | 11.451 | 0.78 (0.68-0.89) | **0.0003** | **0.0009** |
| **Alzheimer’s disease (n=163 cases)** | | | | | |  |  |  |  |  |  |
| Beta-NGF | 0.370 | | 0.98 (0.81-1.18) | 0.8200 | 0.8315 |  | Beta-NGF^a^ | 41.438 | 0.73 (0.55-0.96) | **0.0270** | 0.0570 |
| CCL3 | 1.531 | | 1.11 (0.94-1.31) | 0.2045 | 0.2727 |  | CCL3^b^ | 0.008 | 0.85 (0.69-1.04) | 0.1113 | 0.1615 |
| CCL20 | 1.555 | | 1.17 (1.00-1.38) | 0.0520 | 0.0891 |  | CCL20^b^ | 0.011 | 0.79 (0.64-0.97) | **0.0274** | 0.0570 |
| GDNF | 0.475 | | 1.06 (0.88-1.28) | 0.5480 | 0.5802 |  | GDNF^b^ | 0.822 | 0.79 (0.58-1.08) | 0.1382 | 0.1843 |
| IL-10RA | 0.770 | | 1.13 (0.96-1.33) | 0.1555 | 0.2195 |  | IL-10RA^b^ | 2.193 | 0.84 (0.69-1.03) | 0.0922 | 0.1412 |
| **Vascular dementia (n=195 cases)** | | | | | |  |  |  |  |  |  |
| Beta-NGF | 0.379 | 0.98 (0.83-1.16) | | 0.8471 | 0.8839 |  | Beta-NGF^a^ | 42.098 | 0.90 (0.75-1.08) | 0.2540 | 0.3208 |
| CCL3 | 1.521 | 1.17 (1.00-1.36) | | **0.0473** | 0.0811 |  | CCL3^b^ | 0.008 | 0.79 (0.65-0.96) | **0.0161** | **0.0331** |
| CCL20 | 1.528 | 1.19 (1.02-1.39) | | **0.0260** | 0.0520 |  | CCL20^b^ | 0.011 | 0.75 (0.61-0.92) | **0.0051** | **0.0153** |
| GDNF | 0.507 | 1.16 (0.99-1.35) | | 0.0655 | 0.0983 |  | GDNF^b^ | 0.804 | 0.65 (0.44-0.98) | **0.0387** | 0.0663 |
| IL-10RA | 0.780 | 1.16 (1.00-1.34) | | 0.0501 | 0.0823 |  | IL-10RA^b^ | 11.755 | 0.68 (0.54-0.87) | **0.0019** | **0.0086** |

Abbreviations: SD, standard deviation; CI, confidence interval; FDR, false discovery rate; For biomarker abbreviations, see Supplemental Table 1.

Notes: Numbers printed in bold are statistically significant (p < 0.05).

^*^ Multivariate logistic regression model adjusted for age (continuously), sex, education, physical activity, BMI (categorical), CVD, diabetes, depression, *APOE* genotype.

^†^ P-values corrected for multiple testing by the Benjamini and Hochberg method.

^a^ Modelled with the function x^-1^.

^b^ Modelled with the function x^-2^.

## **Supplemental Table 23**. Associations of IFN-gamma and TNF levels with **all-cause dementia, Alzheimer’s disease and vascular dementia** incidence for participants included in the third wave of measurements (t3) (n=388).

| **Olink Biomarker** | **Value of 1 SD** | **OR (95% CI)  per 1 SD^*^** | **p-value  per 1 SD** |
| --- | --- | --- | --- |
| **All-cause dementia (n=217 cases)** | | | |
| IFN-gamma | 1.033 | 1.12 (0.90-1.39) | 0.3028 |
| TNF | 1.984 | 1.10 (0.88-1.38) | 0.3840 |
| **Alzheimer’s disease (n=60 cases)** | | | |
| IFN-gamma | 0.978 | 1.13 (0.87-1.47) | 0.3664 |
| TNF | 0.984 | 1.15 (0.89-1.49) | 0.2781 |
| **Vascular dementia (n=81 cases)** | | | |
| IFN-gamma | 0.965 | 1.05 (0.84-1.33) | 0.6588 |
| TNF | 0.815 | 0.88 (0.69-1.14) | 0.3361 |

Abbreviations: SD, standard deviation; CI, confidence interval; For biomarker abbreviations, see Supplemental Table 1.

^*^ Multivariate logistic regression model adjusted for age (continuously), sex, education, physical activity, BMI (categorical), CVD, diabetes, depression, *APOE* genotype.
